# Supplementary material for: Hepatic ASPG-mediated lysophosphatidylinositol catabolism impairs insulin signal transduction
Source: EMBO J. 2025 Aug 4;44(18):5005–36. doi: 10.1038/s44318-025-00525-x (PMC12436650; doi:10.1038/s44318-025-00525-x)
Supplement: Supplementary file 1 — Appendix [file 44318_2025_525_MOESM1_ESM.pdf]

**APPENDIX for**

# Hepatic ASPG-mediated lysophosphatidylinositol catabolism impairs systemic insulin signal transduction

## Table of contents

Appendix Table S1 ....................................................................2
Appendix Table S2 ....................................................................2
Appendix Table S3 ....................................................................3
Appendix Table S4 ....................................................................5
Appendix Table S5 ....................................................................5

**Appendix Table S1. Clinical characteristics of human cohort 1 study**

| **Human cohort 1** | **n=69** |
|---|---|
| Male/Female | 55/14 |
| Age (years) | 56.43 ± 13.71 |
| BMI (kg/m$^2$) | 23.87 ± 3.44 |
| AST(U/L) | 29.88 ± 12.44 |
| ALT(U/L) | 34.49 ± 25.88 |
| Fasting Glucose (mmol/L) | 6.18 ± 2.42 |
| Serum Triglyceride (mmol/L) | 1.93 ± 1.69 |
| Serum Insulin (mU/L) | 14.35 ± 11.75 |

Data are presented as the mean ± SD.

**Appendix Table S2. Clinical characteristics of the patients with MASLD**

| **MASLD** | **HOMA-IR< 2** | **HOMA-IR≥ 2** | **P** |
|---|---|---|---|
| Number | 14 | 18 | / |
| Age (years) | 52.64 ± 9.18 | 56.56 ± 11.47 | / |
| Male/Female | 13/1 | 17/1 | / |
| BMI (kg/m$^2$) | 24.34 ± 3.13 | 26.72 ± 3.69 | 0.063 |
| Glucose (mmol/L) | 4.12 ± 0.26 | 8.57 ± 1.61 | <0.001 |
| Serum Triglyceride (mmol/L) | 1.26 ± 0.57 | 2.40 ± 1.93 | 0.042 |
| AST(U/L) | 35.71 ± 12.00 | 54.56 ± 28.65 | 0.028 |
| ALT(U/L) | 32.71 ± 16.59 | 58.89 ± 44.59 | 0.046 |
| Serum Insulin (mU/L) | 8.20 ± 2.82 | 22.79 ± 5.65 | <0.001 |

Data are presented as the mean ± SD. P: unpaired two-tailed Student's t-test.

**Appendix Table S3. Primer sequences for qRT-PCR.**

| Gene | Sequence (5'-3') |
|---|---|
| Human Aspg | F: AACCGGGCAACCAAGGTAG |
| | R: CCAGCTCCCTGTTGATTGTGA |
| Human Pnpla7 | F: GGAAAAGCGTGATGGTTGC |
| | R: GAGCAGGTCCTTCTTGGCA |
| Human Pla2g6 | F: CATCACAGCCGTATCATCAGC |
| | R: TCGGTGACATCCATCTGAGTG |
| Human Enpp2 | F:ACAACGAGGAGAGCTGCAAT |
| | R:AGAAGTCCAGGCTGGTGAGA |
| Human Abdh16a | F:TGCCGTTTTCTCACTATGCTG |
| | R:CCGATGTGTTGCTTCCAAGATG |
| Human Abdh12 | F:GTCCTGGAATACAGGCCAAAC |
| | R: CAATGGTCACGTCTTCCTCTG |
| Human Pnpla6 | F:TCCTCCCTCGTGGATACCTC |
| | R:GTGGGCGTCTCTTTCTGGA |
| Human Pnpla8 | F:TCCTTCTGCGATACCTGATGT |
| | R:CTCGCTGAAGAGATAAACGCTT |
| Human Ddhd1 | F:CCTGCCTCAGTGACGAGAAC |
| | R:AAGCCGGGTACGTTTCCTTTC |
| Human Ddhd2 | F:ATGTCATCAGTGCAGTCACAAC |
| | R:ACTGGTTCATACAAGCTGCCA |
| Human Lypla1 | F:CCTTTGCAGGTATCAGAAGTTCA |
| | R:GCTGCCTGTTTAATCCCAGAT |
| Human Pla1a | F:CAGACACCGACAATTTGGGTA |
| | R:CCAGGGCGCTGATGTAGAG |
| Human Sepp1 | F:GAAGACCTGCGAGTAAAACTGA |
| | R:ACAAGACGGCCACATCTATCATA |
| Human β-actin | F:CATGTACGTTGCTATCCAGGC |
| | R:CTCCTTAATGTCACGCACGAT |
| Mouse Sepp1 | F:AGCTCTGCTTGTTACAAAGCC |
| | R:CAGGTCTTCCAATCTGGATGC |
| Mouse FetuinA | F:ATCCGCTCCACAAGGTACAG |
| | R:GGTCCAAAGCATGGCAAGT |
| Mouse FetuinB | F:CTCCGACTTCTGGTGCTCTG |
| | R:CAGGGCAAATCCTGCAACTG |
| Mouse Lect2 | F:CCCTGTCAGGAACTTTTCATCC |
| | R:CGGTAGTAAACACCGTTCAGGT |
| Mouse Fgl1 | F:CCCACAACAATCCTCATTTCAGC |
| | R:ACACCTGGGTGATGCCTTTG |
| Mouse Aspg | F:AGGCGGAGTATTGGTACCTG |
| | R:TGTGGGCCTGTGCATACTCTT |
| Mouse Fasn | F:GGAGGTGGTGATAGCCGGTAT |
| | R:TGGGTAATCCATAGAGCCCAG |

| | |
|---|---|
| Mouse Dgat2 | F:GCGCTACTTCCGAGACTACTT<br>R:GGGCCTTATGCCAGGAAACT |
| Mouse Acc2 | F:CGCTCACCAACAGTAAGGTGG<br>R:GCTTGGCAGGGAGTTCCTC |
| Mouse Cd36 | F:ATGGGCTGTGATCGGAACTG<br>R:TTTGCCACGTCATCTGGGTTT |
| Mouse Fabp1 | F:ATGAACTTCTCCGGCAAGTACC<br>R:CTGACACCCCCTTGATGTCC |
| Mouse Gpat1 | F:ACAGTTGGCACAATAGACGTTT<br>R:CCTTCCATTTCAGTGTTGCAGA |
| Mouse Cpt1a | F:CTCCGCCTGAGCCATGAAG<br>R:CACCAGTGATGATGCCATTCT |
| Mouse Scd1 | F:TTCTTGCGATACACTCTGGTGC<br>R:CGGGATTGAATGTTCTTGTCGT |
| Mouse Pck1 | F:CTGCATAACGGTCTGGACTTC<br>R:CAGCAACTGCCCGTACTCC |
| Mouse Pcx | F:CTGAAGTTCCAAACAGTTCGAGG<br>R:CGCACGAAACACTCGGATG |
| Mouse G6pc1 | F:CGACTCGCTATCTCCAAGTGA<br>R:GTTGAACCAGTCTCCGACCA |
| Mouse Il-6 | F:TAGTCCTTCCTACCCCAATTTCC<br>R:TTGGTCCTTAGCCACTCCTTC |
| Mouse Tnfα | F:CCCTCACACTCAGATCATCTTCT<br>R:GCTACGACGTGGGCTACAG |
| Mouse Nf-kb | F:AGGCTTCTGGGCCTTATGTG<br>R:TGCTTCTCTCGCCAGGAATAC |
| Mouse Col1a1 | F:GCTCCTCTTAGGGGCCACT<br>R:CCACGTCTCACCATTGGGG |
| Mouse Col3a1 | F:CTGTAACATGGAAACTGGGGAAA<br>R: CCATAGCTGAACTGAAAACCACC |
| Mouse Pla2g6 | F:GCAAGCTGATTACCAGGAAGG<br>R:GAGAGAAGAGGGGGTGAGTTG |
| Mouse Gpr55 | F: CTGGCAGTCCATATCCCCAC<br>R: GCACCAGCAGTAAATCGAAAACA |
| Mouse Mboat7 | F: TACCGCACCTACCTGGATTG<br>R: AGAAGACCGGGATCATGTAGAA |
| Mouse Rplp0 | F:GCTTCGGCAGCACATATACTAAAAT<br>R:CGCTTCACGAATTTGCGTGTCAT |

**Appendix Table S4.    Target sequences for shRNA and siRNA.**

| Gene | Sequence (5'-3') |
|---|---|
| Mouse shFoxo1 | CCGCCAAACACCAGTCTAAAT |
| Mouse Ptp1b siRNA | CCAGGACAUUCGACAUGAAUU |
| Mouse shSepp1 | GGTGTCAGAACACATCGCA |

**Appendix Table S5.    Primer sequences for ChIP-qPCR.**

| Promoter | Sequence(5'-3') |
|---|---|
| Mouse Aspg （-2008~-1803） | F:TCTCCTGGGATTAAAGGCCTG<br>R:GGACCCTGTCTCAACAAAACCA |
| Mouse Aspg （-1729~-1494） | F:CTGGCTGATCTTCCTTCTTCCTTG<br>R:TGCTACCAGATGCAAGGACG |
| Mouse Aspg （-1006~-784） | F:GCTCATACTGCCTGACATGTTTG<br>R:GCGGTCAAGTTCTTGTTCAGGGA |
