# Supplementary figures and images for: Hepatic ASPG-mediated lysophosphatidylinositol catabolism impairs insulin signal transduction
Source: EMBO J. 2025 Aug 4;44(18):5005–36. doi: 10.1038/s44318-025-00525-x (PMC12436650; doi:10.1038/s44318-025-00525-x)

1E

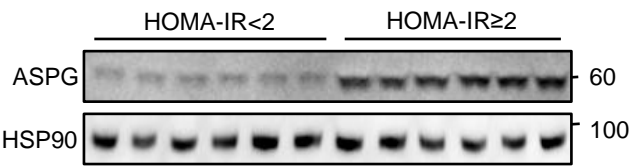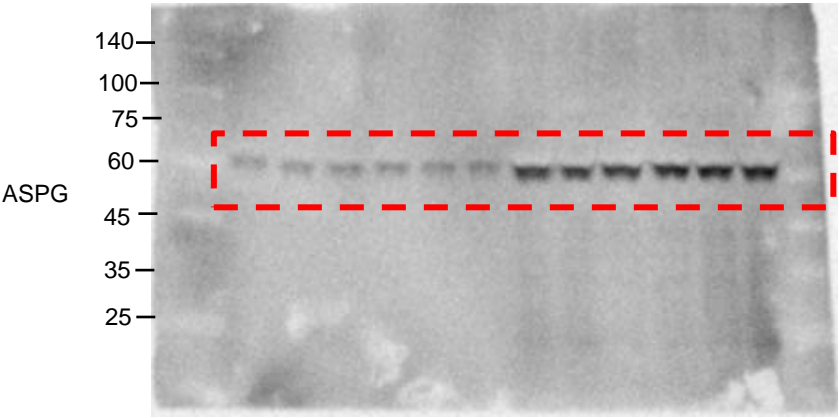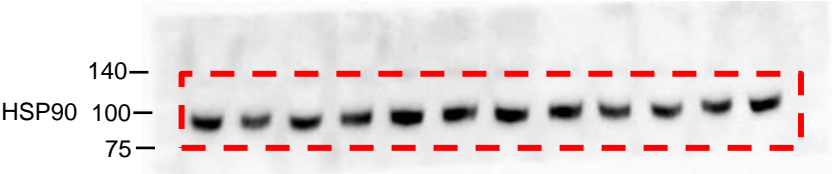

Supplement: Supplementary file 3 — Source data Fig. 1 [file 44318_2025_525_MOESM3_ESM.zip › SD Figure 1/1E/1E.pdf]

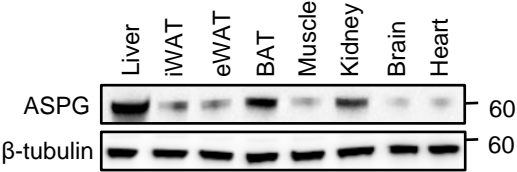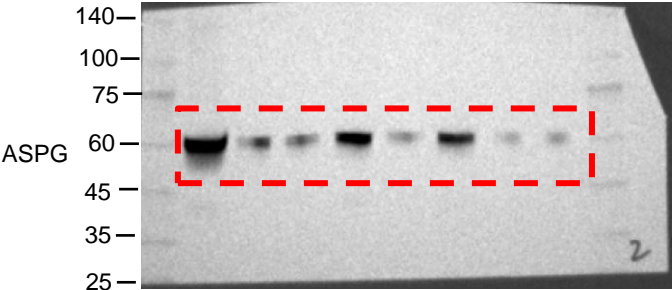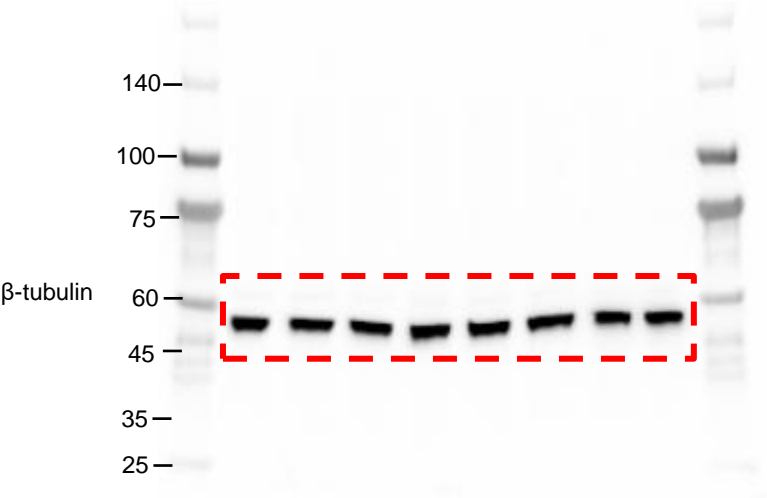

Supplement: Supplementary file 3 — Source data Fig. 1 [file 44318_2025_525_MOESM3_ESM.zip › SD Figure 1/1G/1G.pdf]

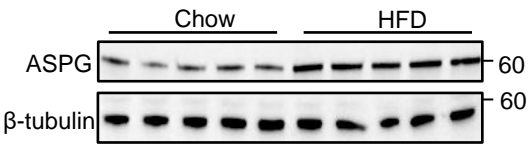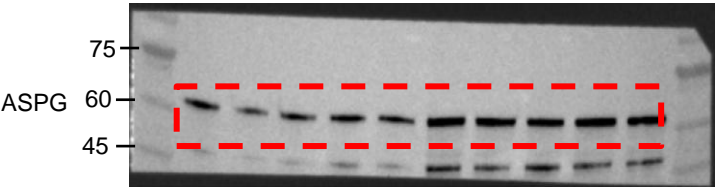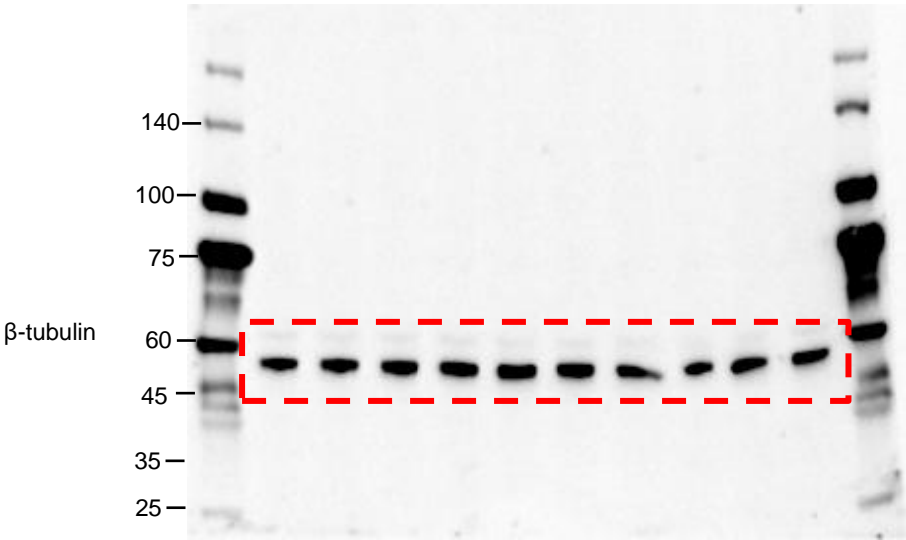

Supplement: Supplementary file 3 — Source data Fig. 1 [file 44318_2025_525_MOESM3_ESM.zip › SD Figure 1/1H/1H.pdf]

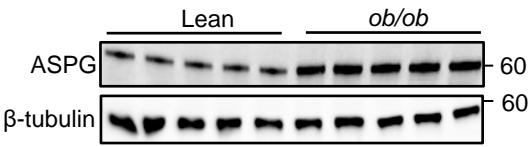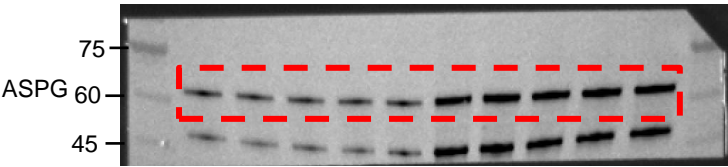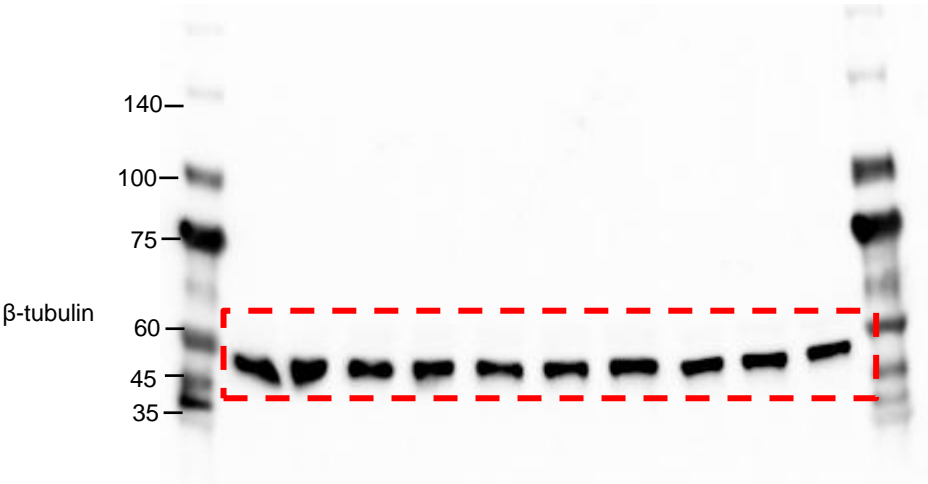

Supplement: Supplementary file 3 — Source data Fig. 1 [file 44318_2025_525_MOESM3_ESM.zip › SD Figure 1/1I/1I.pdf]

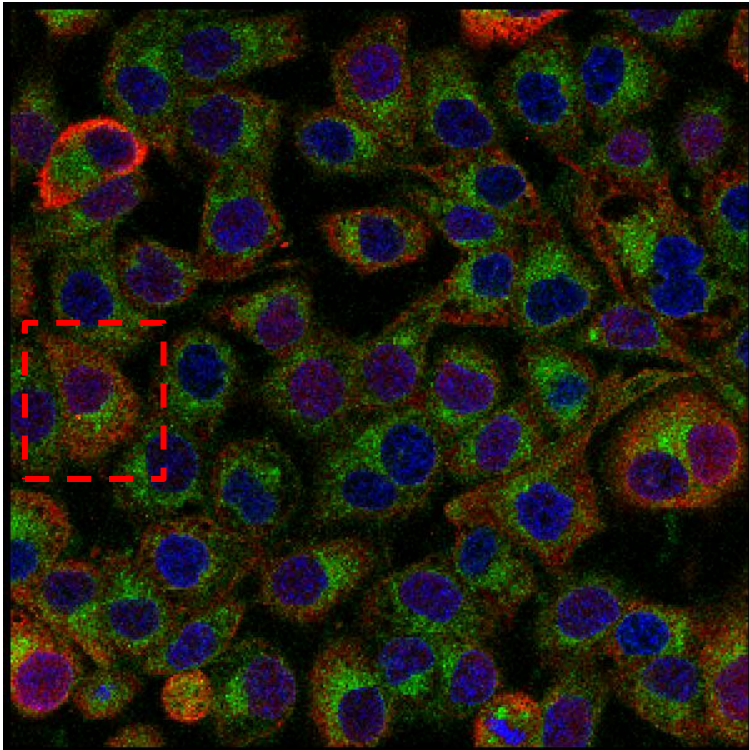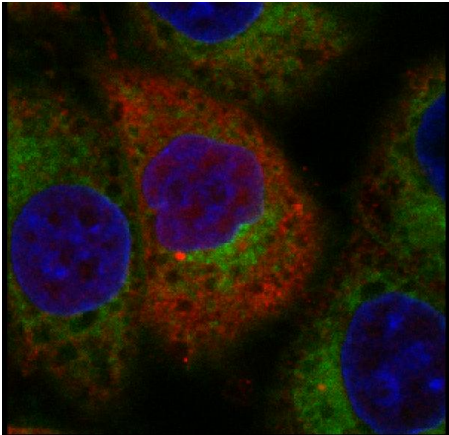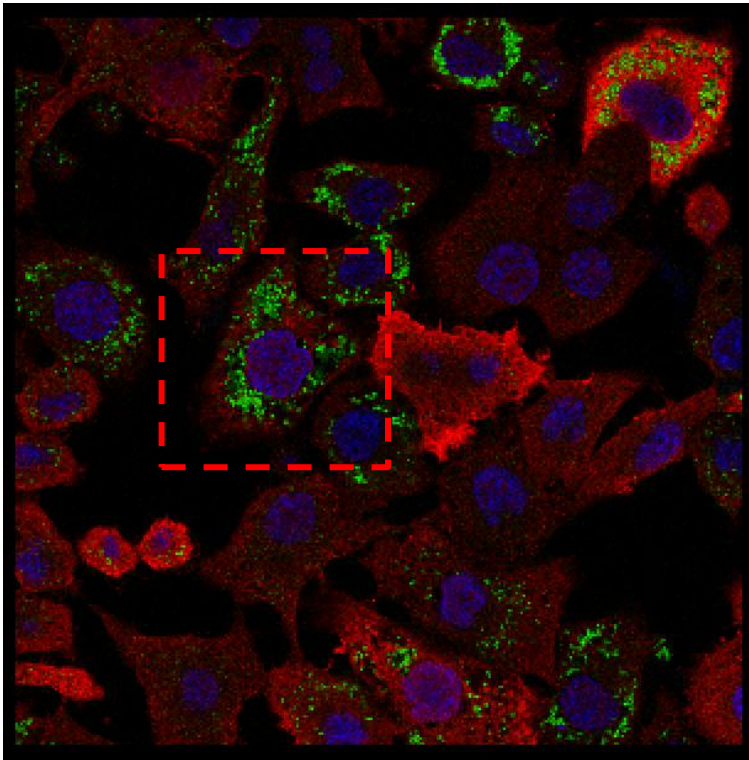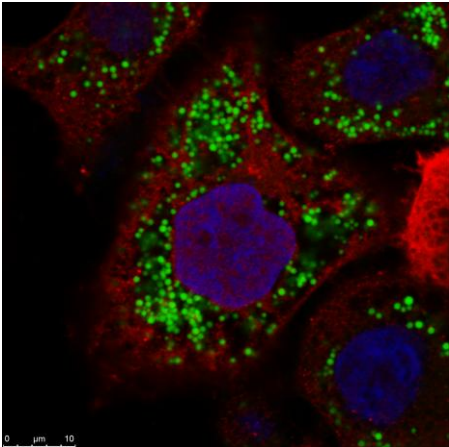

Supplement: Supplementary file 3 — Source data Fig. 1 [file 44318_2025_525_MOESM3_ESM.zip › SD Figure 1/1J/1J.pdf]

1K

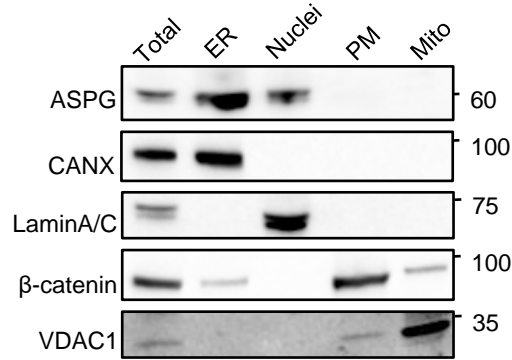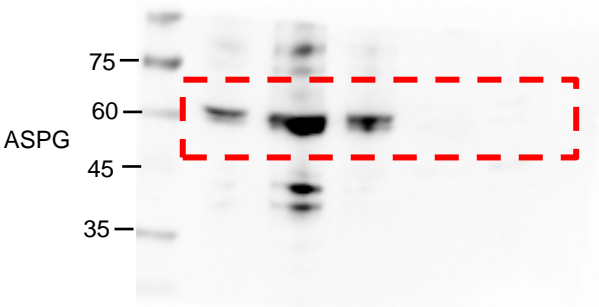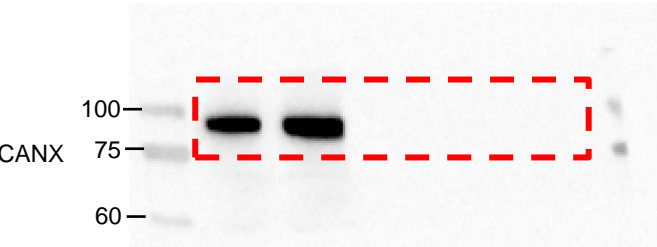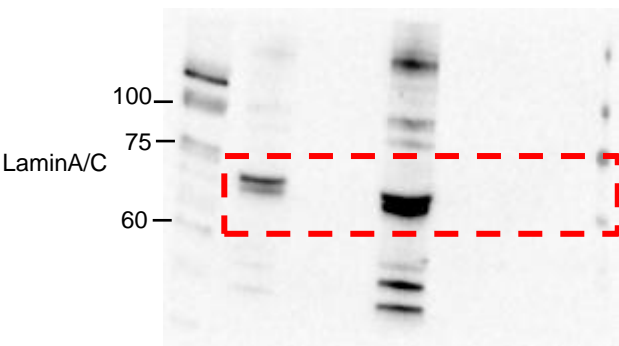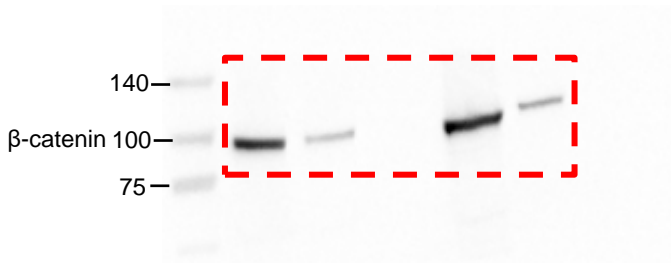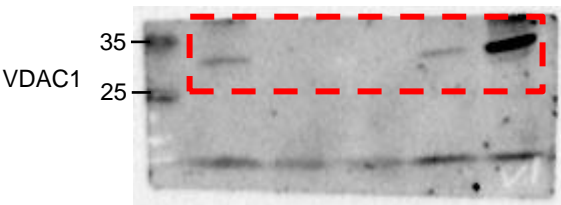

Supplement: Supplementary file 3 — Source data Fig. 1 [file 44318_2025_525_MOESM3_ESM.zip › SD Figure 1/1K/1K.pdf]

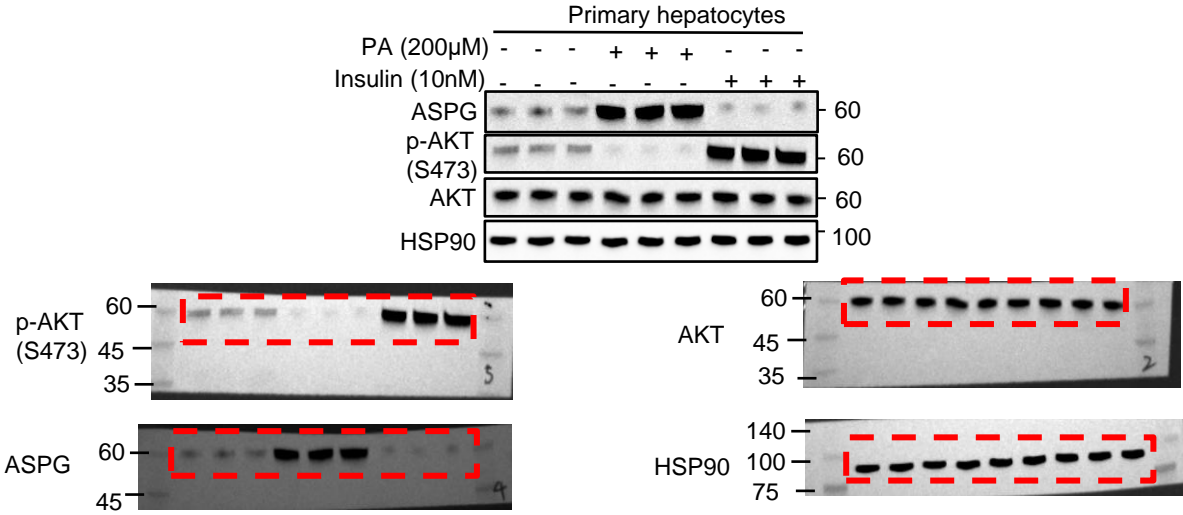

Supplement: Supplementary file 4 — Source data Fig. 2 [file 44318_2025_525_MOESM4_ESM.zip › SD Figure 2/2A/2A.pdf]

2B

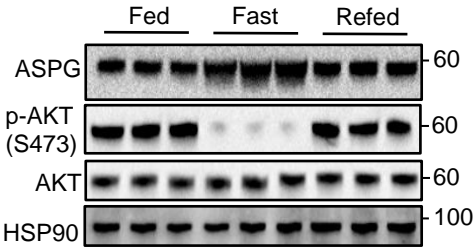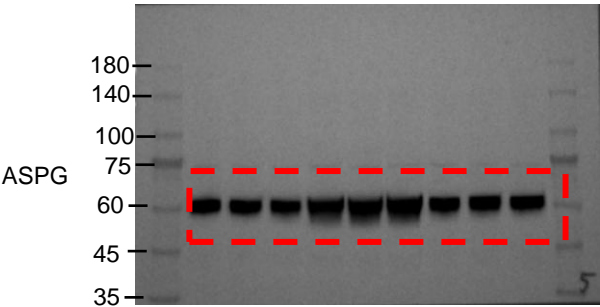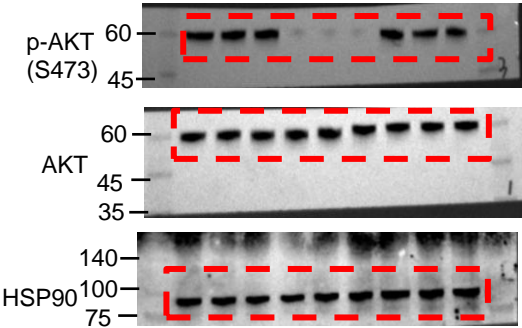

e

Supplement: Supplementary file 4 — Source data Fig. 2 [file 44318_2025_525_MOESM4_ESM.zip › SD Figure 2/2B/2B.pdf]

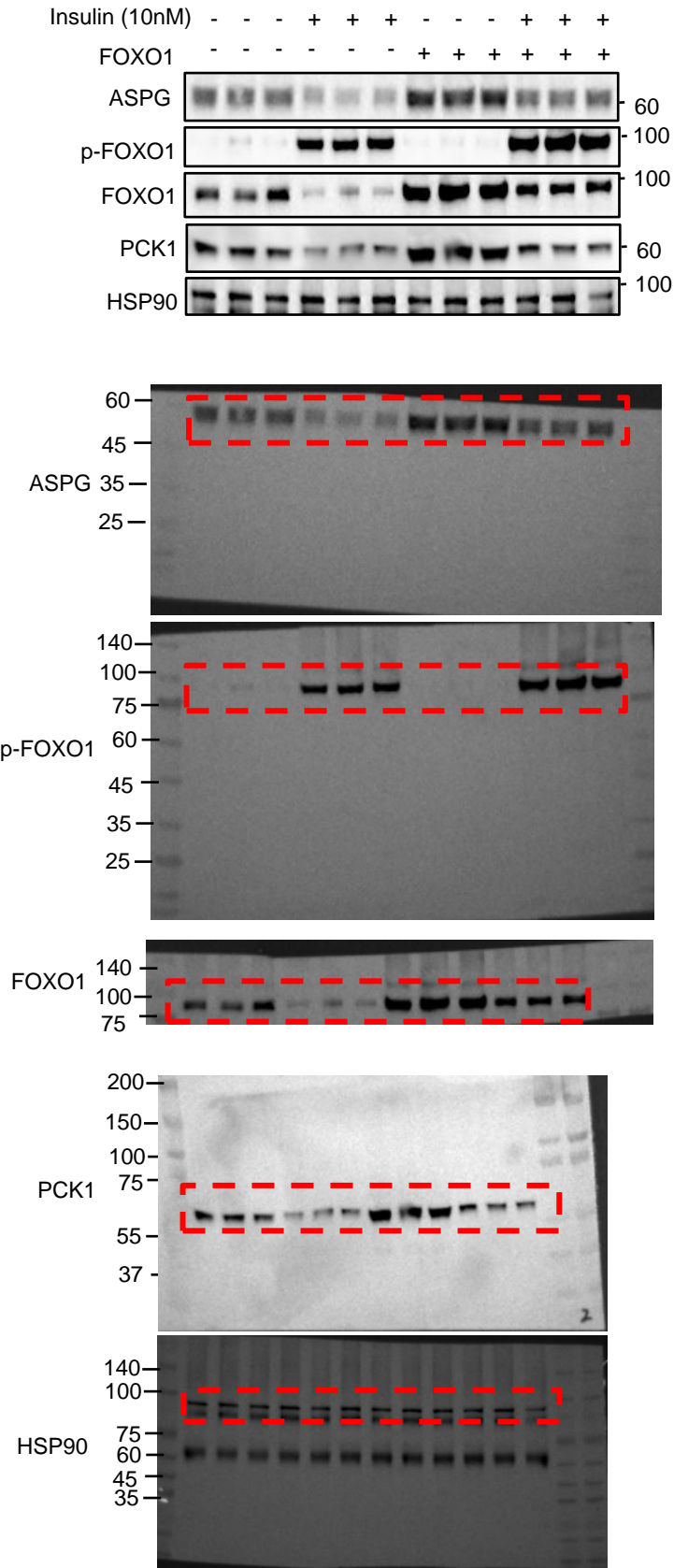

Supplement: Supplementary file 4 — Source data Fig. 2 [file 44318_2025_525_MOESM4_ESM.zip › SD Figure 2/2E/2E.pdf]

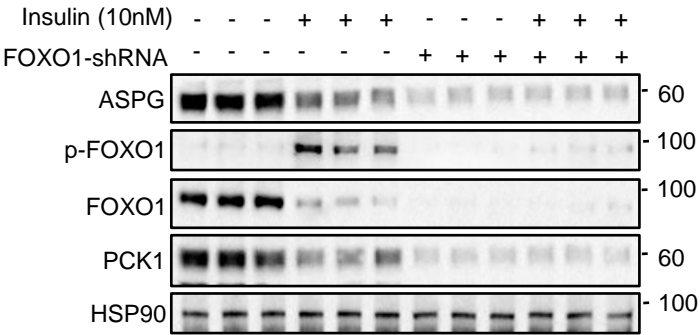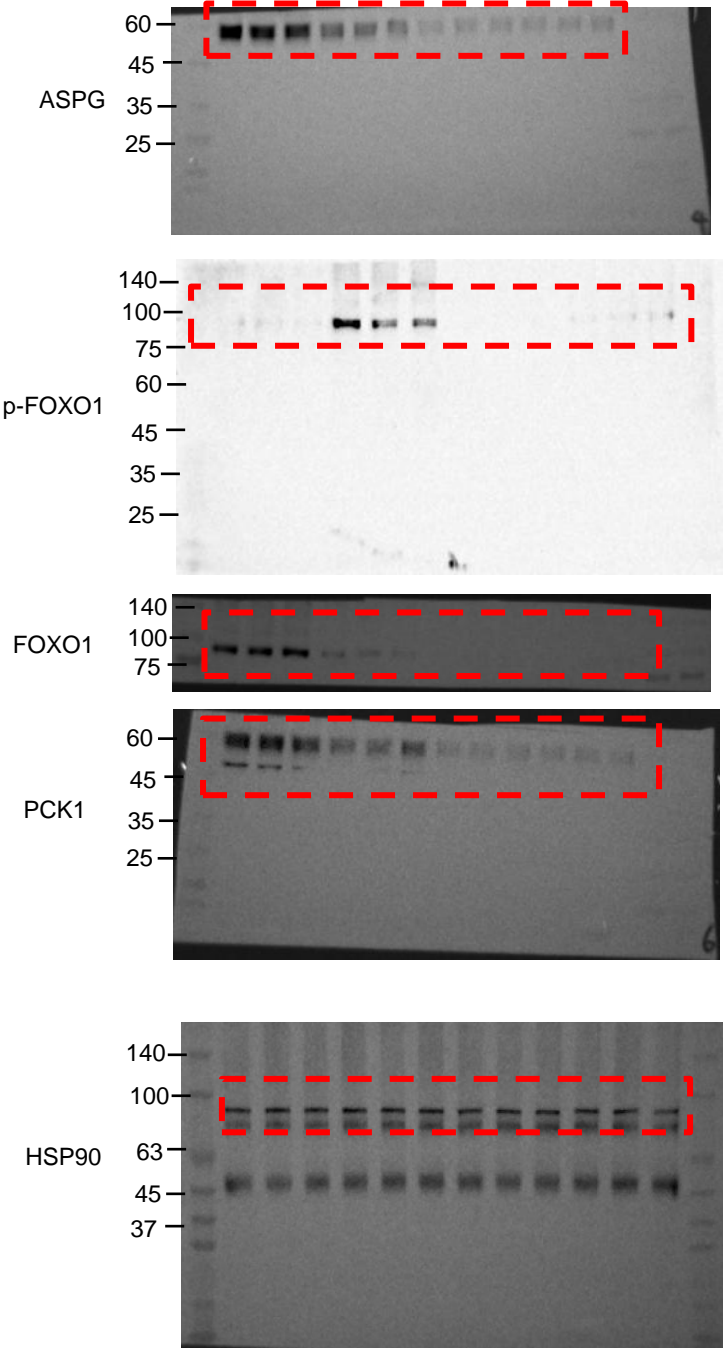

Supplement: Supplementary file 4 — Source data Fig. 2 [file 44318_2025_525_MOESM4_ESM.zip › SD Figure 2/2F/2F.pdf]

3B

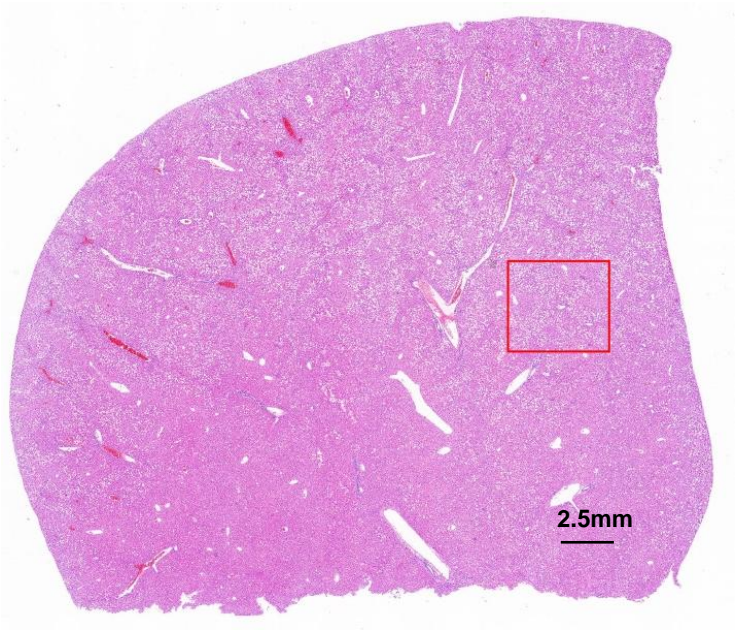

WT

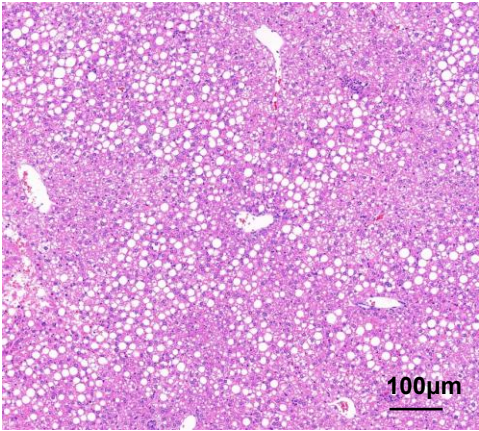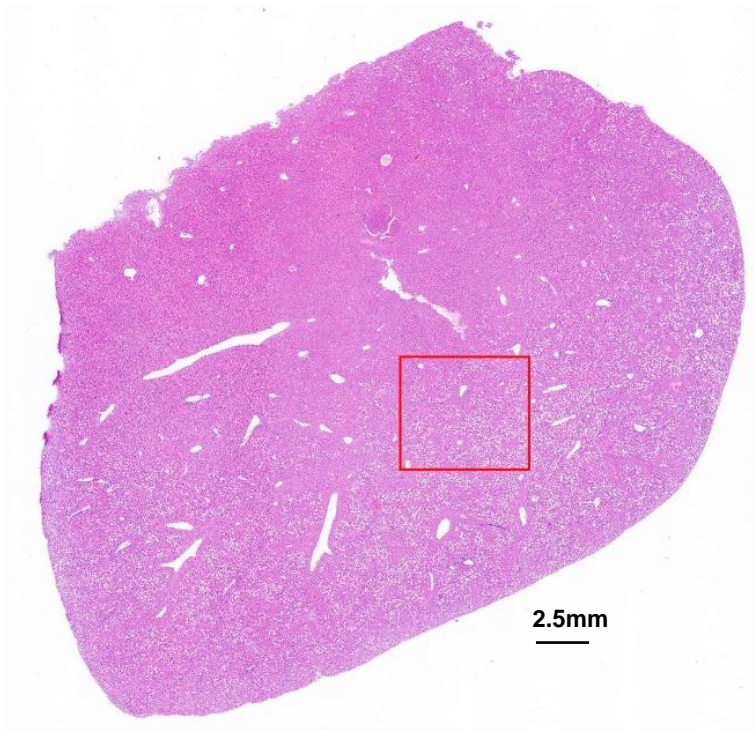

LKO

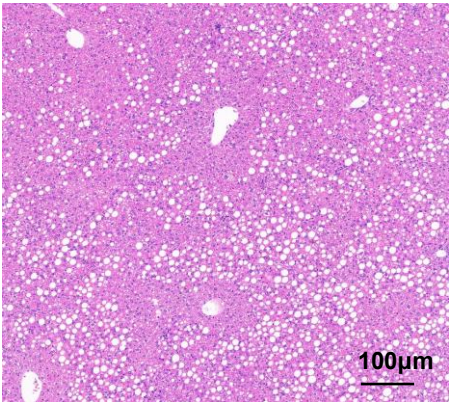

Supplement: Supplementary file 5 — Source data Fig. 3 [file 44318_2025_525_MOESM5_ESM.zip › SD Figure 3/3B/3B.pdf]

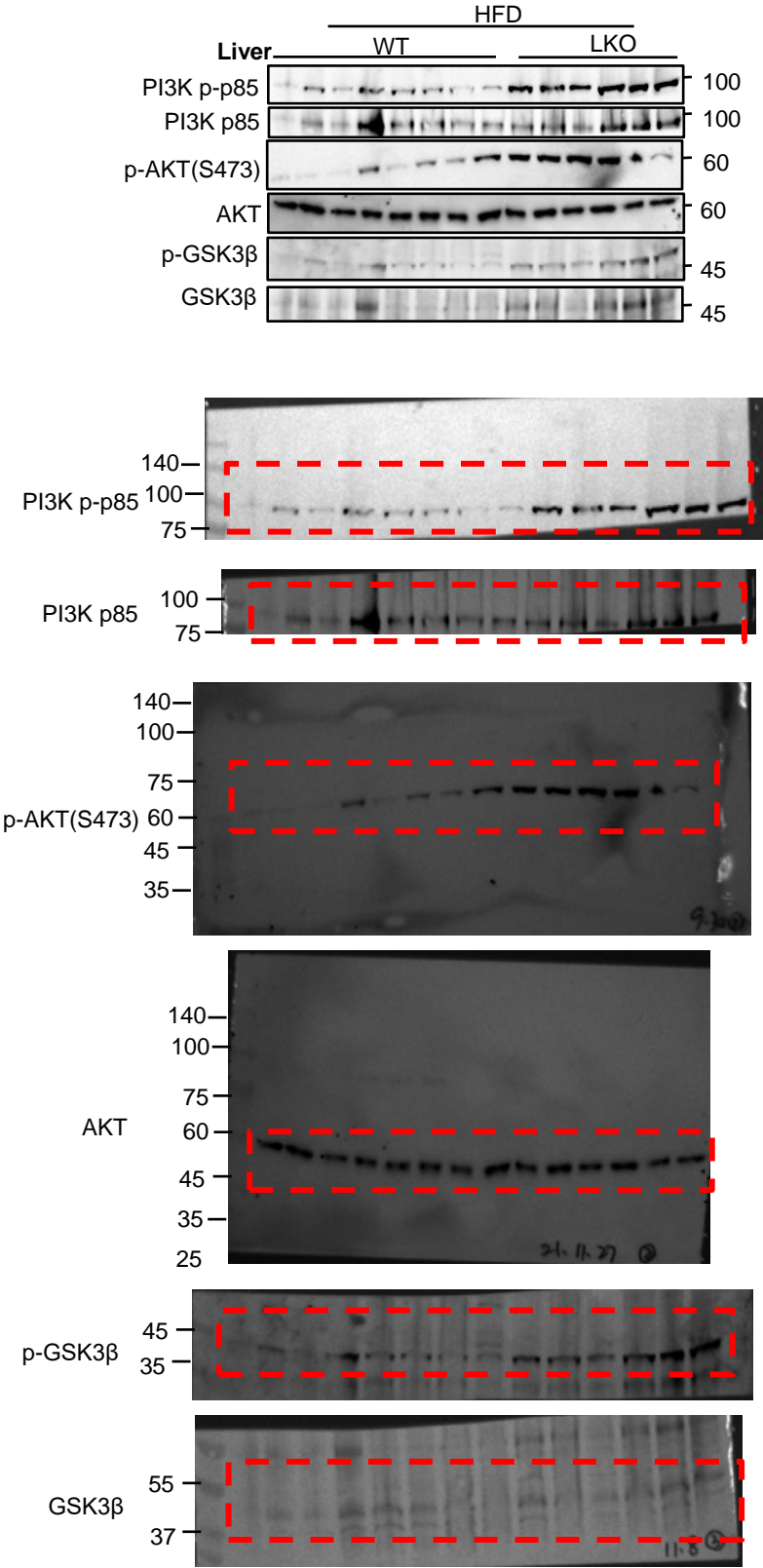

Supplement: Supplementary file 5 — Source data Fig. 3 [file 44318_2025_525_MOESM5_ESM.zip › SD Figure 3/3O/3O.pdf]

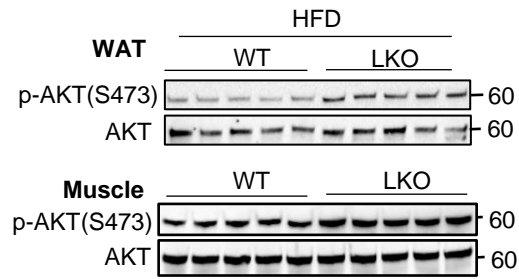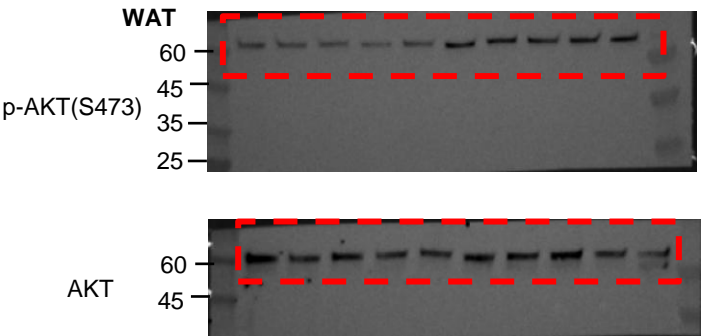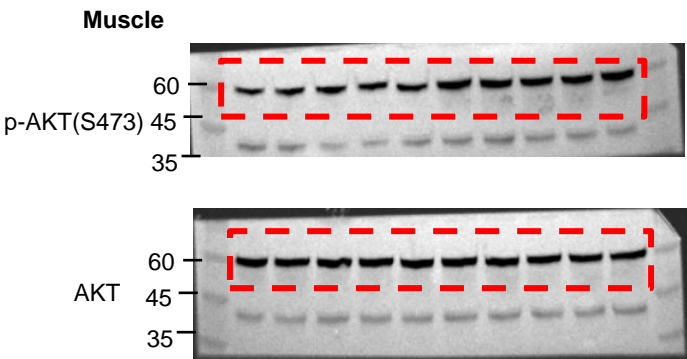

Supplement: Supplementary file 5 — Source data Fig. 3 [file 44318_2025_525_MOESM5_ESM.zip › SD Figure 3/3P/3P.pdf]

4E

WT  
+AAV8-VEC

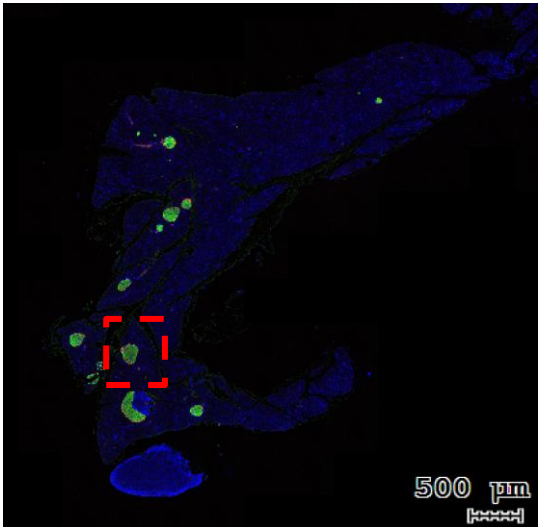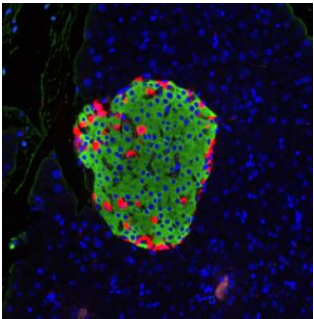

WT  
+AAV8-ASPG

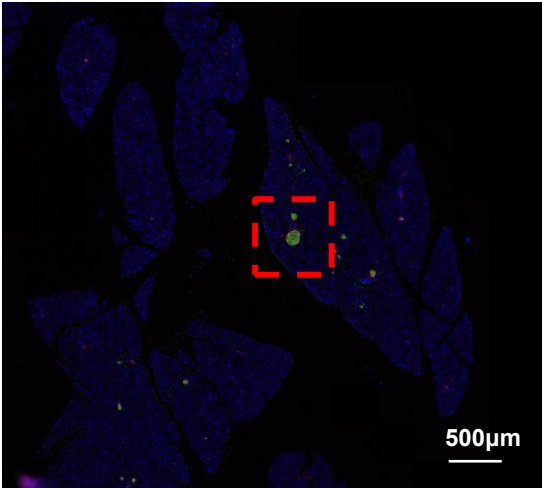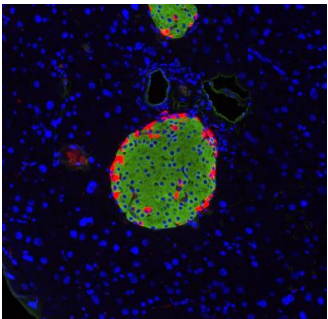

LKO  
+AAV8-VEC

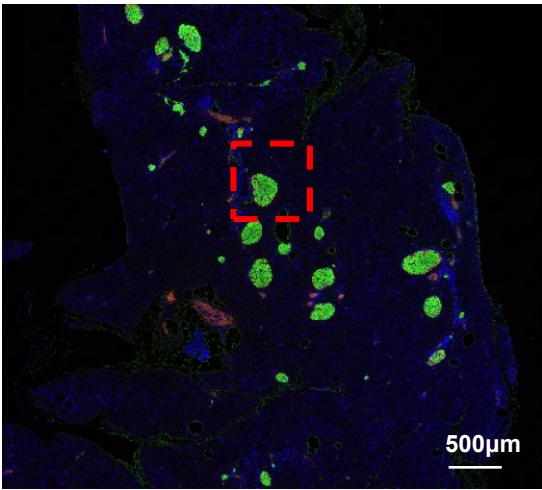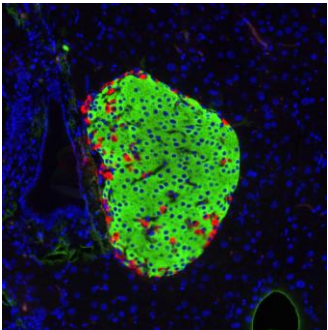

LKO  
+AAV8-ASPG

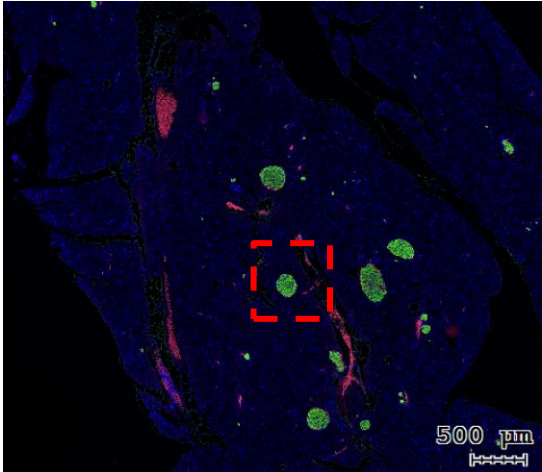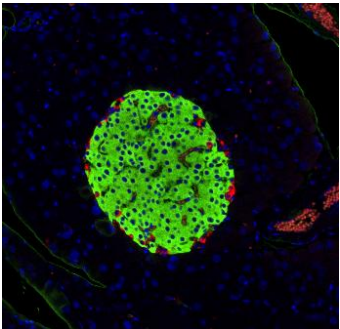

Supplement: Supplementary file 6 — Source data Fig. 4 [file 44318_2025_525_MOESM6_ESM.zip › SD Figure 4/4E/4E.pdf]

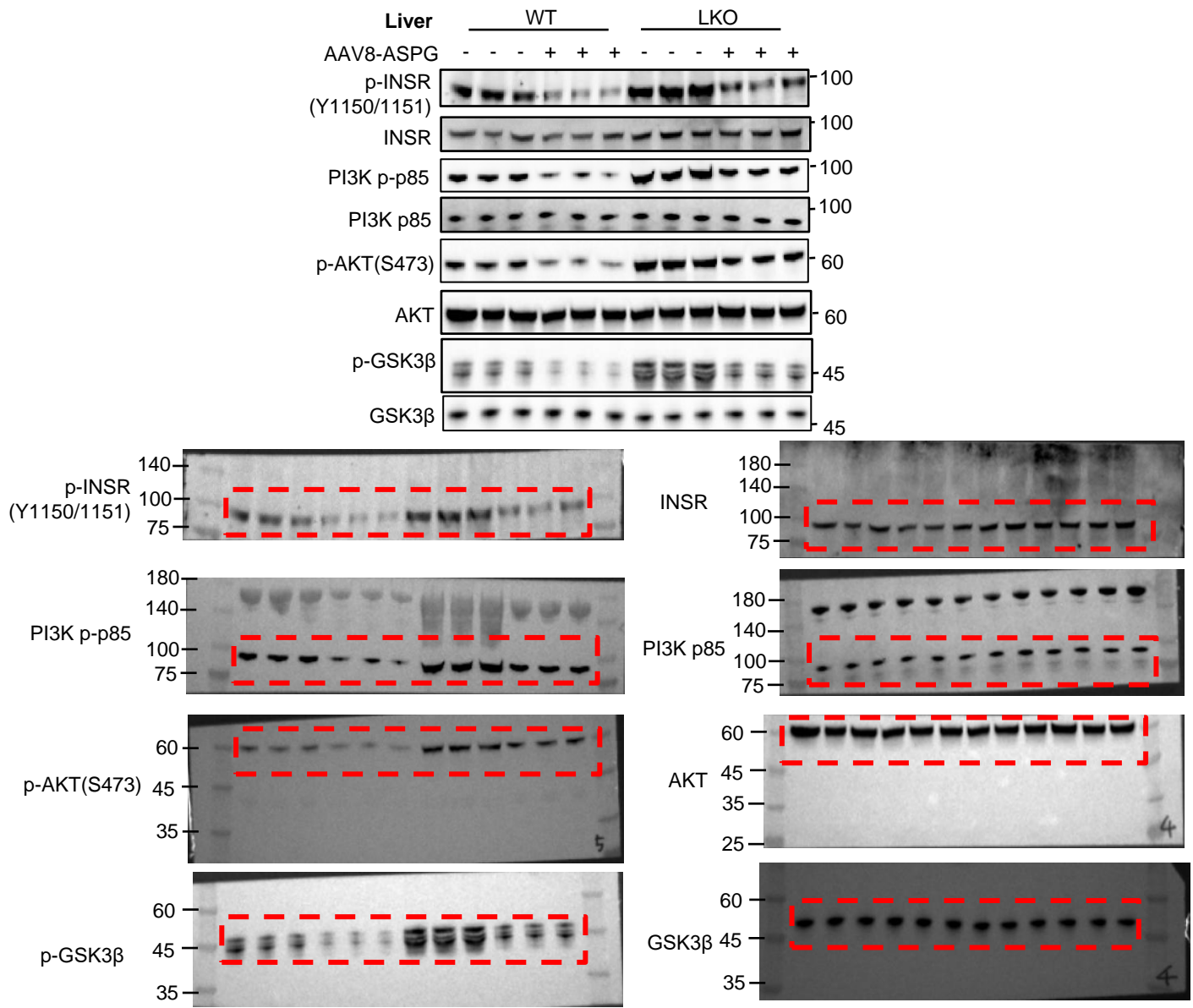

Supplement: Supplementary file 6 — Source data Fig. 4 [file 44318_2025_525_MOESM6_ESM.zip › SD Figure 4/4H/4H.pdf]

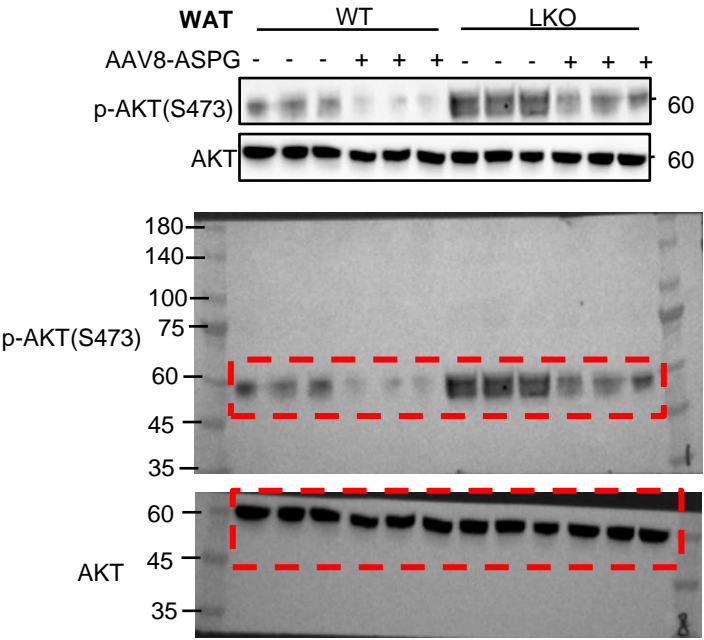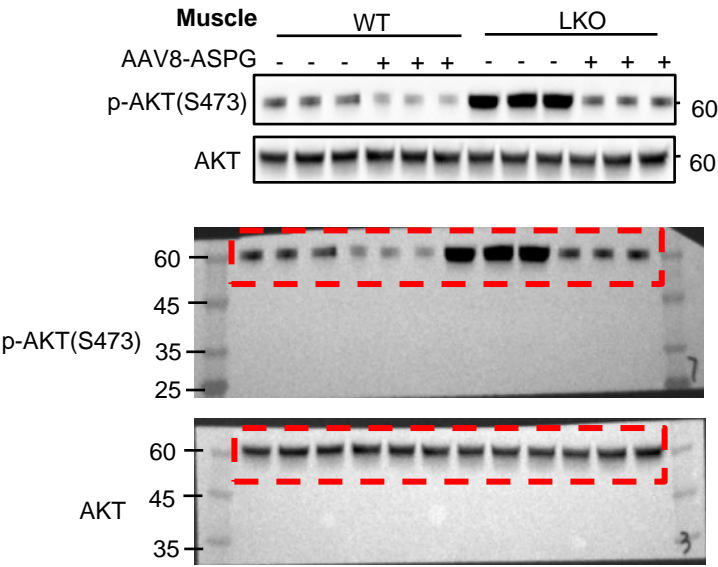

Supplement: Supplementary file 6 — Source data Fig. 4 [file 44318_2025_525_MOESM6_ESM.zip › SD Figure 4/4I/4I.pdf]

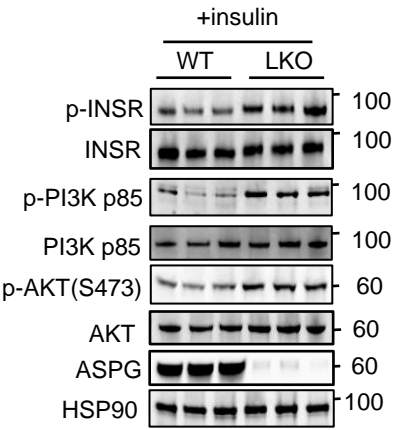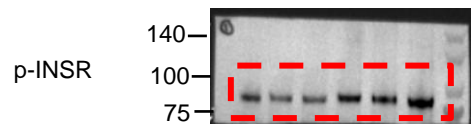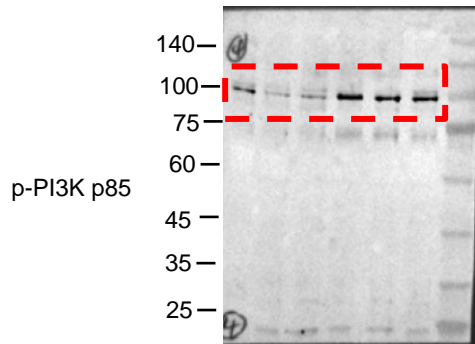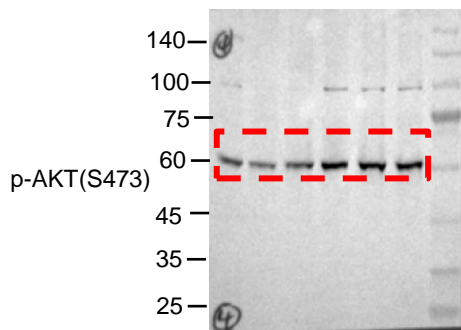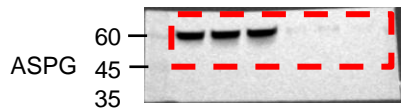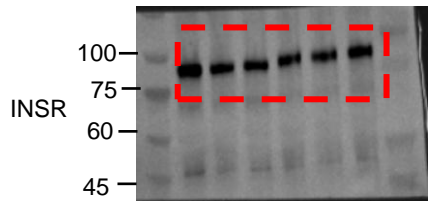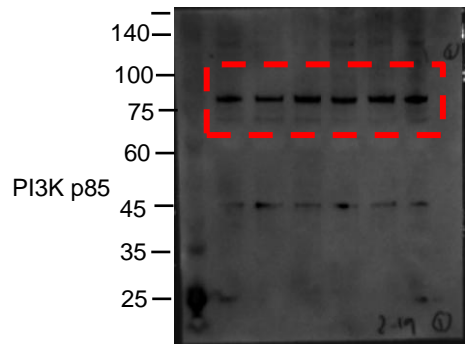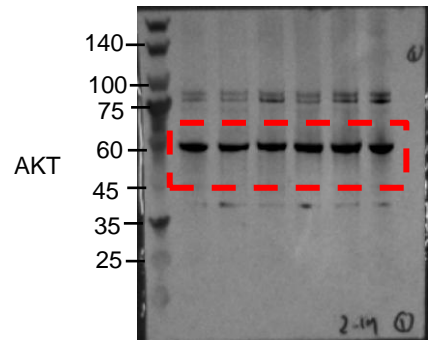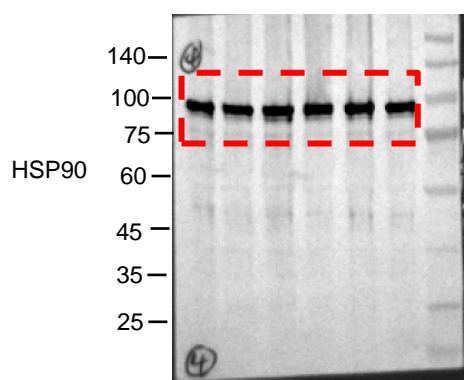

Supplement: Supplementary file 7 — Source data Fig. 5 [file 44318_2025_525_MOESM7_ESM.zip › SD Figure 5/5A/5A.pdf]

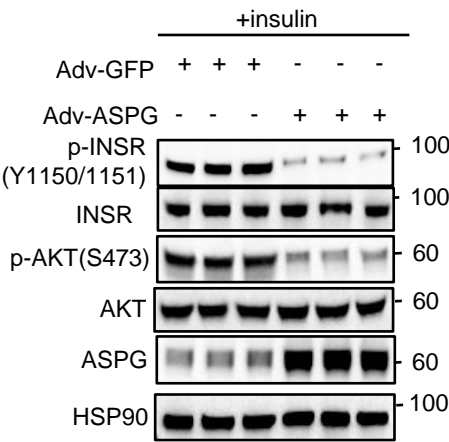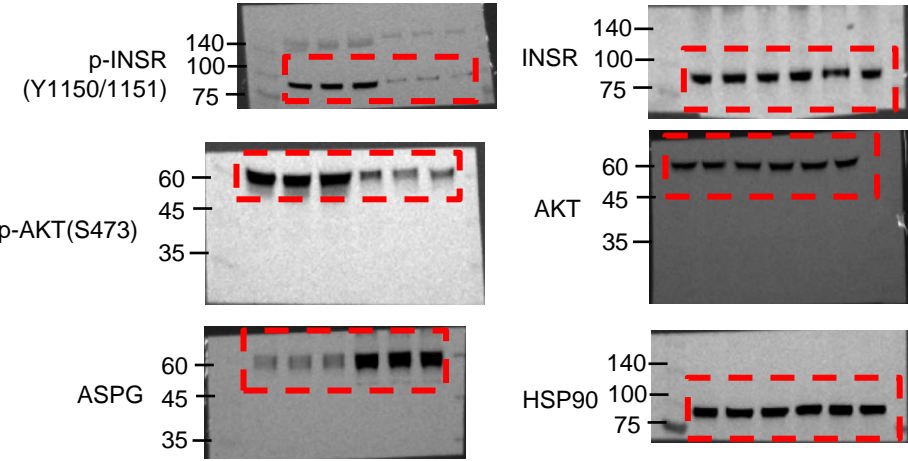

Supplement: Supplementary file 7 — Source data Fig. 5 [file 44318_2025_525_MOESM7_ESM.zip › SD Figure 5/5B/5B.pdf]

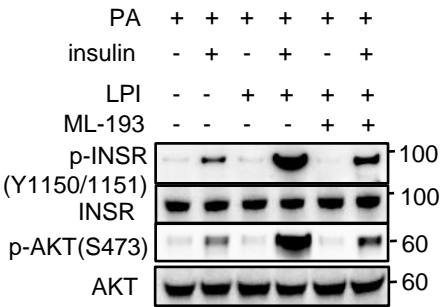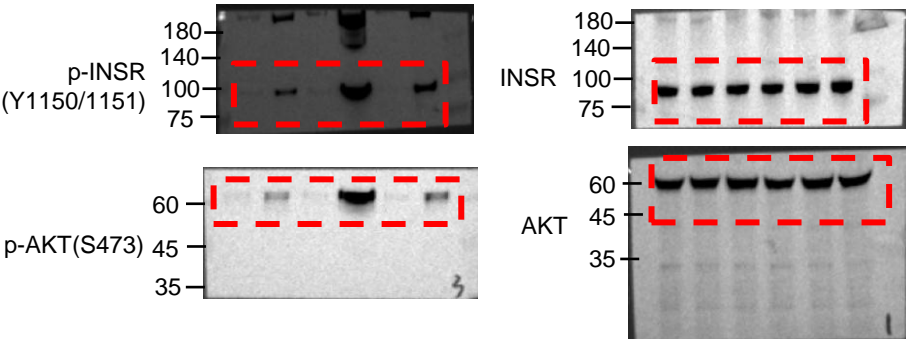

Supplement: Supplementary file 7 — Source data Fig. 5 [file 44318_2025_525_MOESM7_ESM.zip › SD Figure 5/5F/5F.pdf]

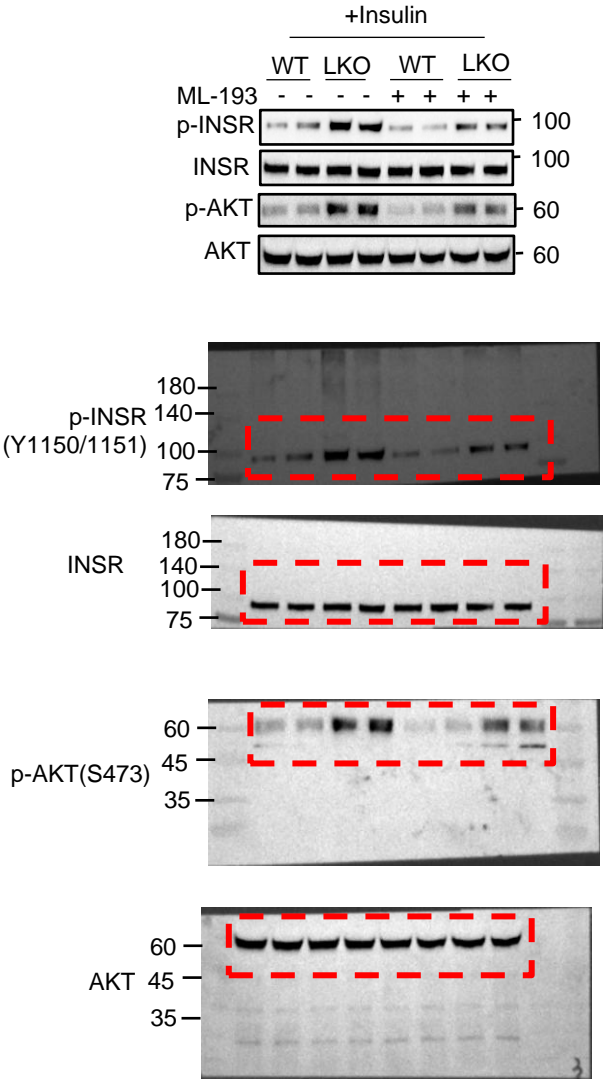

Supplement: Supplementary file 7 — Source data Fig. 5 [file 44318_2025_525_MOESM7_ESM.zip › SD Figure 5/5G/5G.pdf]

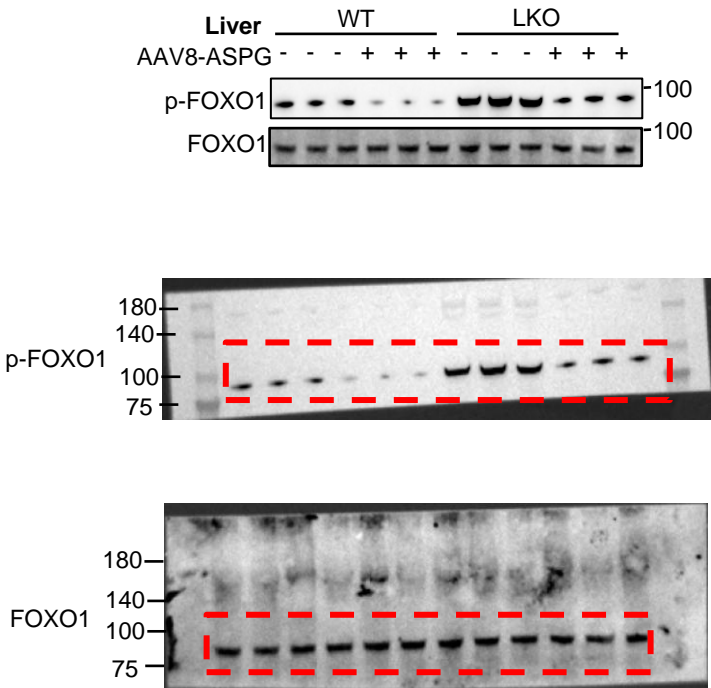

Supplement: Supplementary file 8 — Source data Fig. 6 [file 44318_2025_525_MOESM8_ESM.zip › SD Figure 6/6F/6F.pdf]

7H

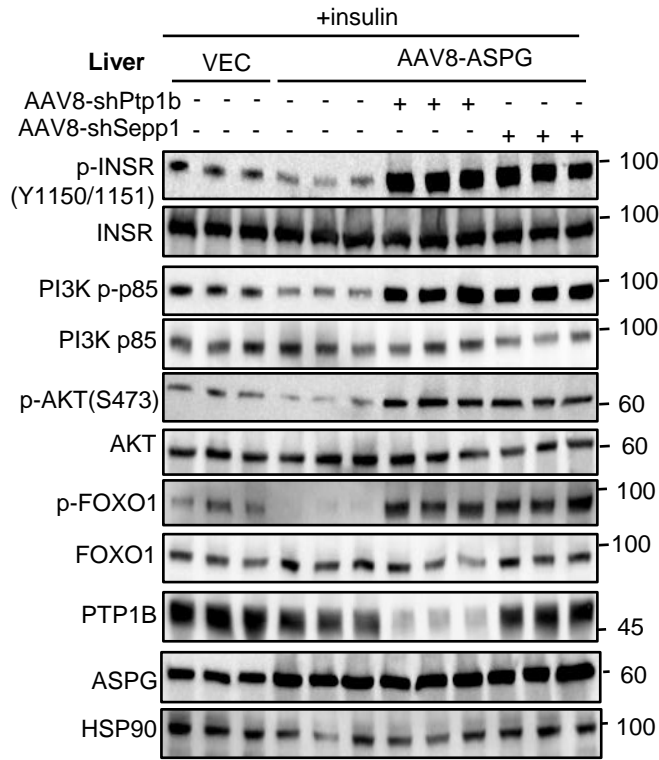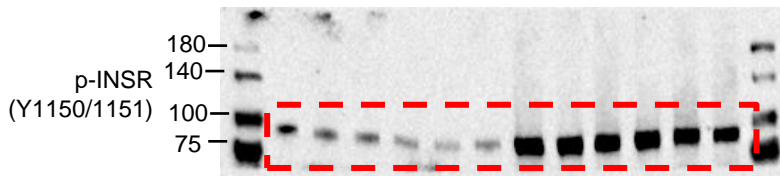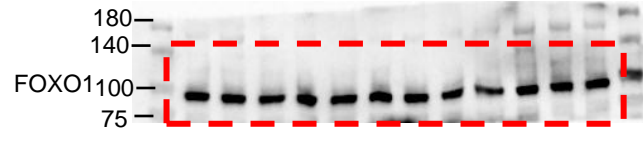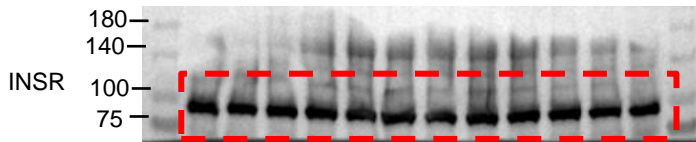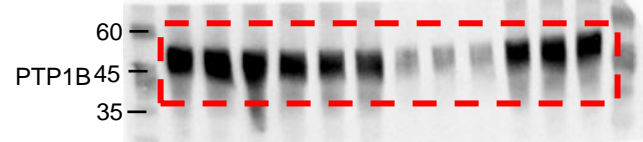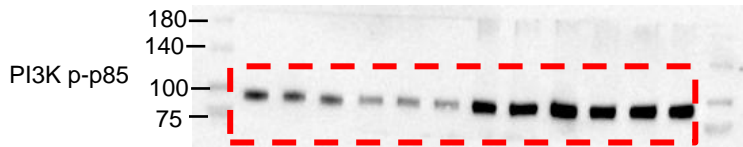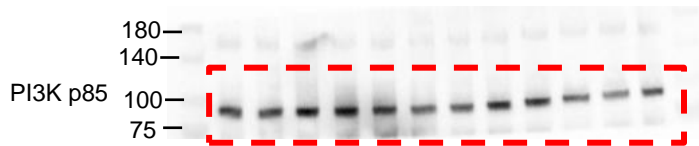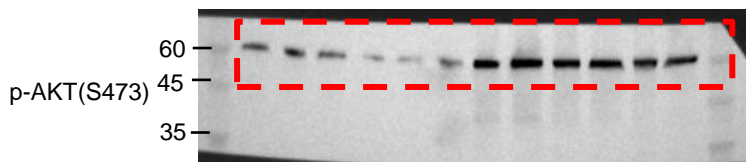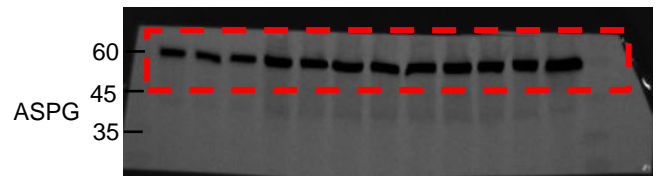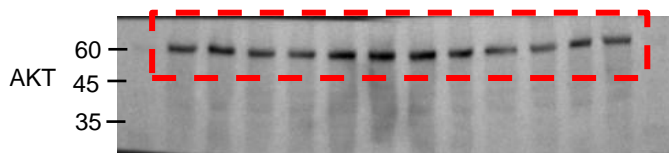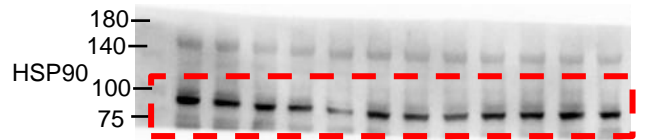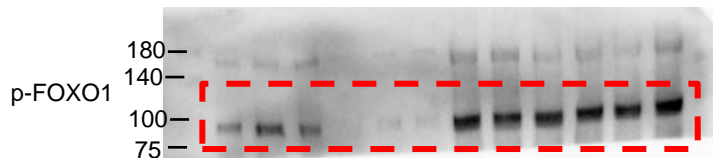

Supplement: Supplementary file 9 — Source data Fig. 7 [file 44318_2025_525_MOESM9_ESM.zip › SD Figure 7/7H/7H.pdf]

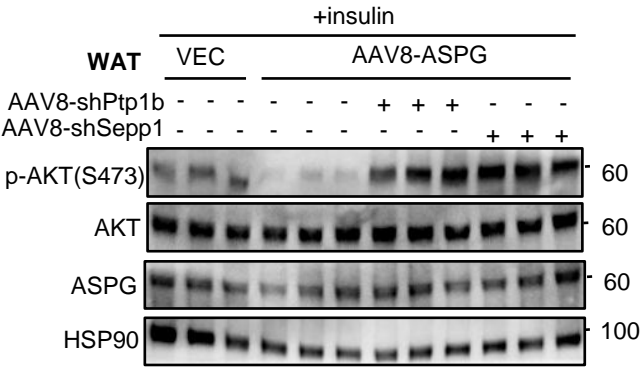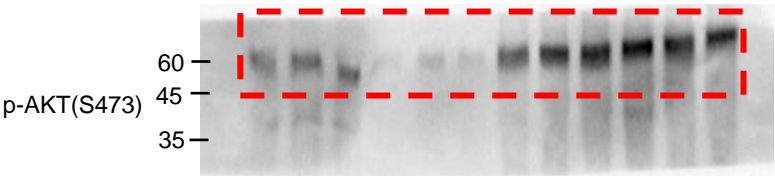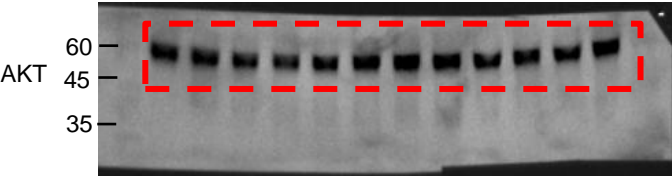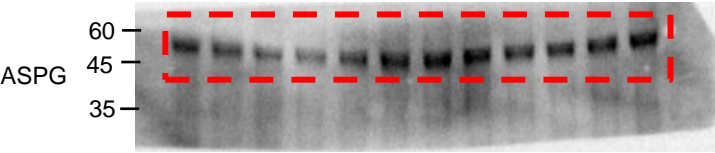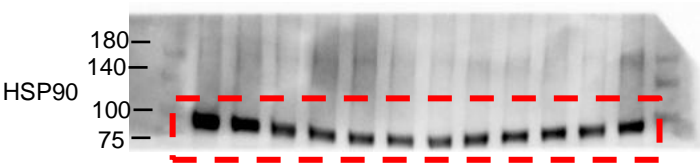

Supplement: Supplementary file 9 — Source data Fig. 7 [file 44318_2025_525_MOESM9_ESM.zip › SD Figure 7/7I/7I.pdf]

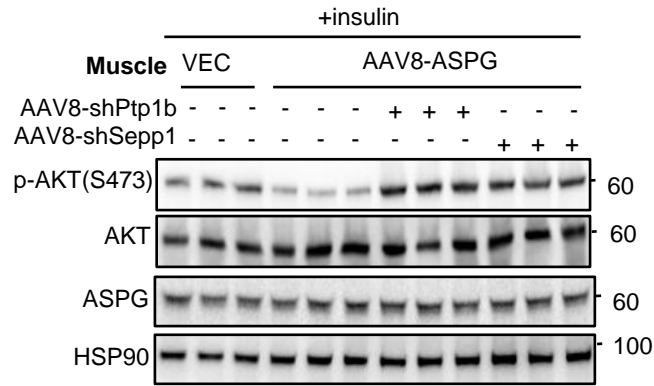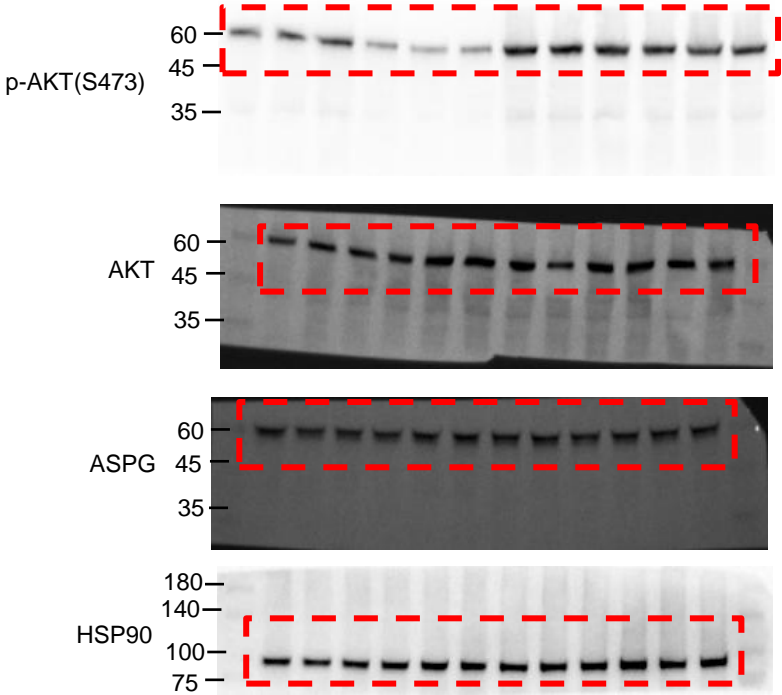

Supplement: Supplementary file 9 — Source data Fig. 7 [file 44318_2025_525_MOESM9_ESM.zip › SD Figure 7/7J/7J.pdf]

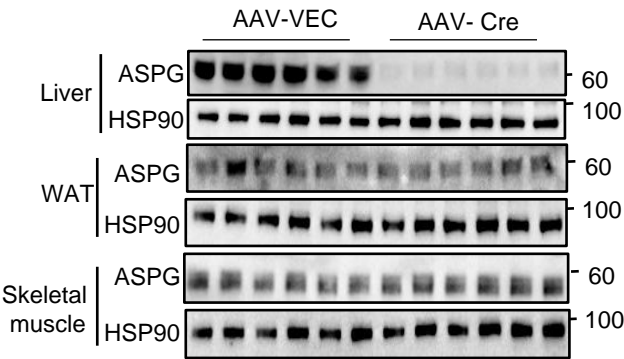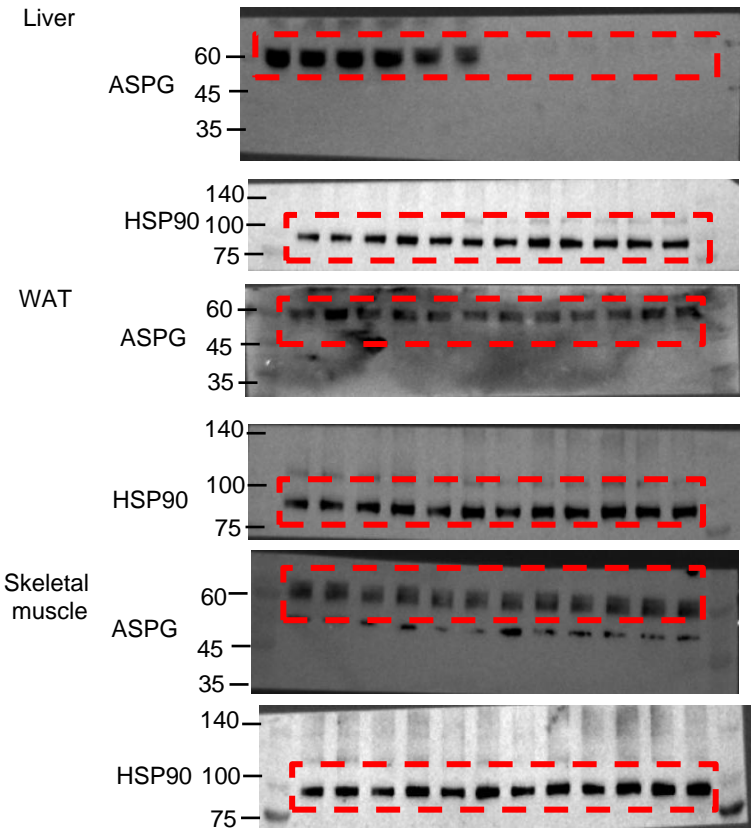

Supplement: Supplementary file 10 — Source data Fig. 8 [file 44318_2025_525_MOESM10_ESM.zip › SD Figure 8/8B/8B.pdf]

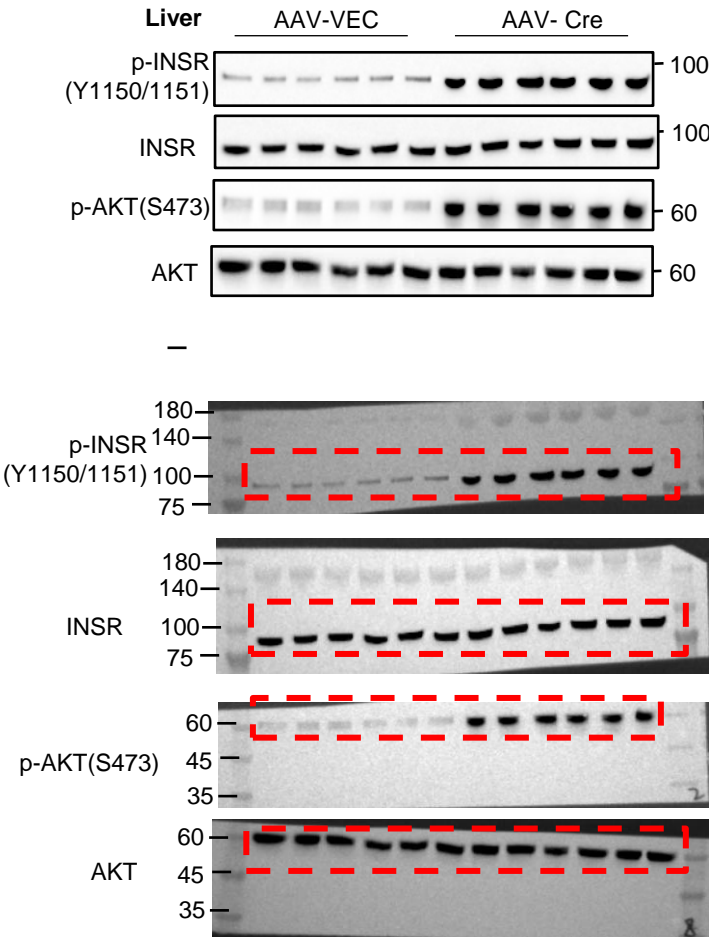

Supplement: Supplementary file 10 — Source data Fig. 8 [file 44318_2025_525_MOESM10_ESM.zip › SD Figure 8/8J/8J.pdf]

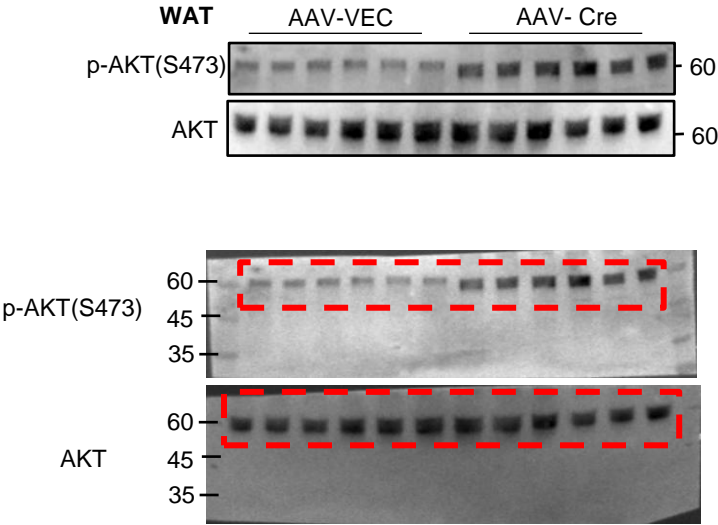

Supplement: Supplementary file 10 — Source data Fig. 8 [file 44318_2025_525_MOESM10_ESM.zip › SD Figure 8/8K/8K.pdf]

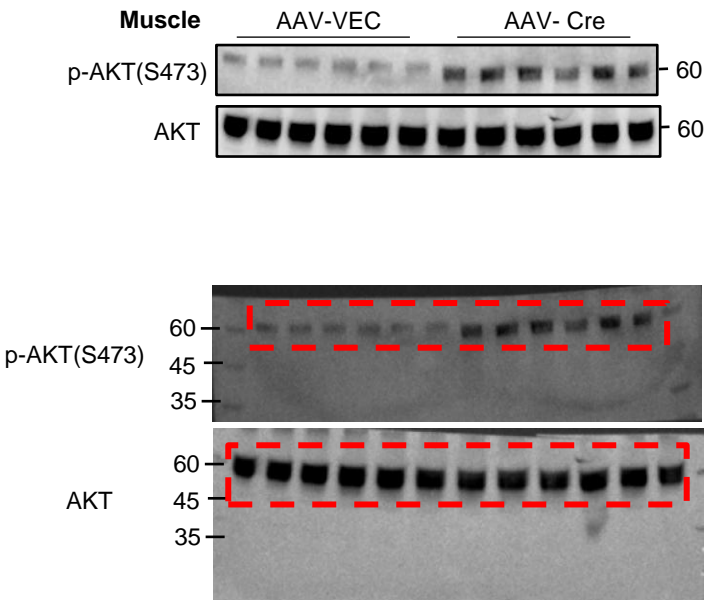

Supplement: Supplementary file 10 — Source data Fig. 8 [file 44318_2025_525_MOESM10_ESM.zip › SD Figure 8/8L/8L.pdf]

EV3B

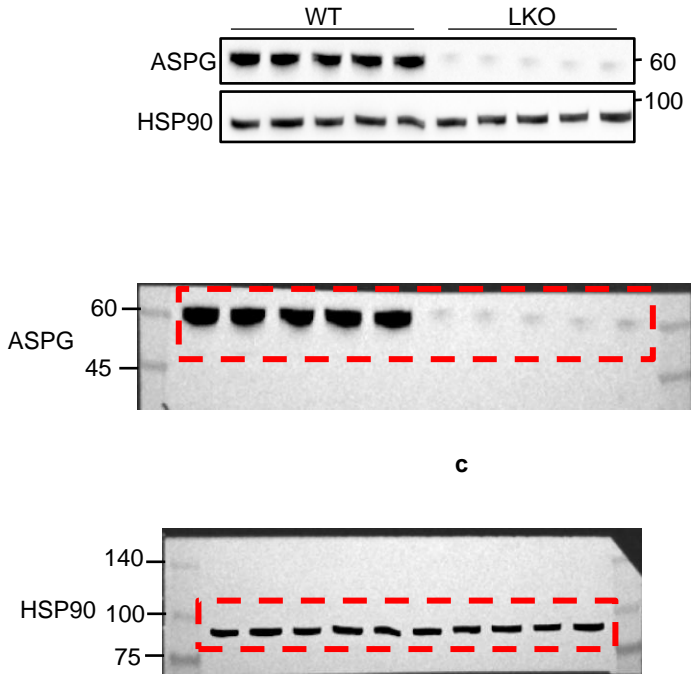

Supplement: Supplementary file 11 — Figure EV Source Data [file 44318_2025_525_MOESM11_ESM.zip › supp figures/Figure EV3/3B.pdf]

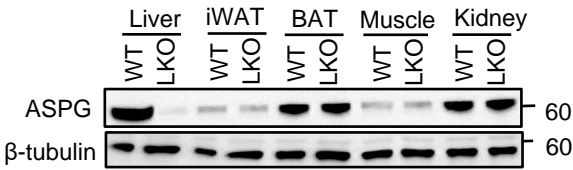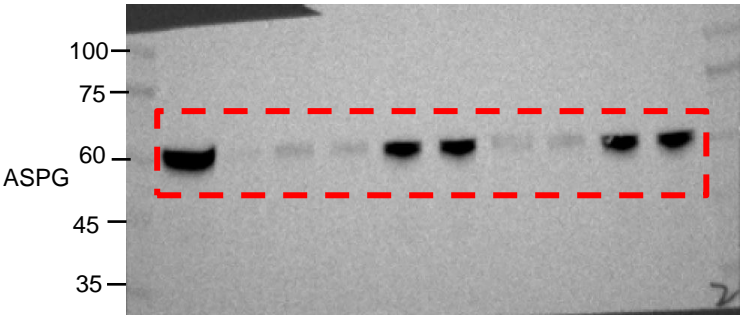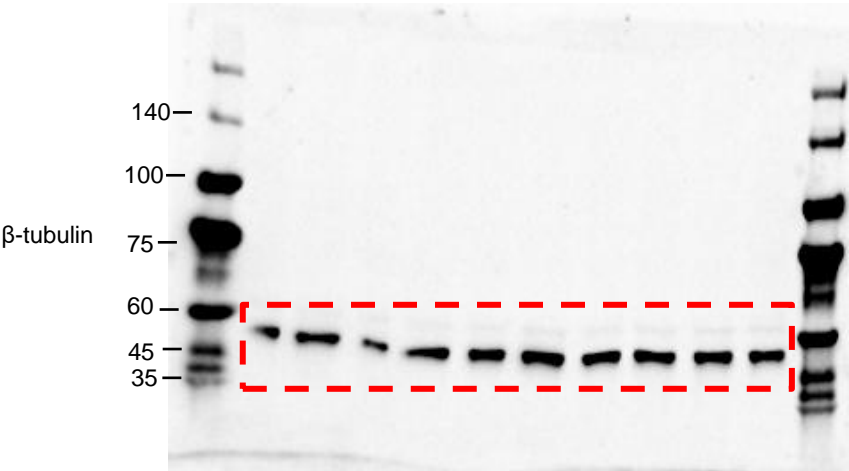

Supplement: Supplementary file 11 — Figure EV Source Data [file 44318_2025_525_MOESM11_ESM.zip › supp figures/Figure EV3/3C.pdf]

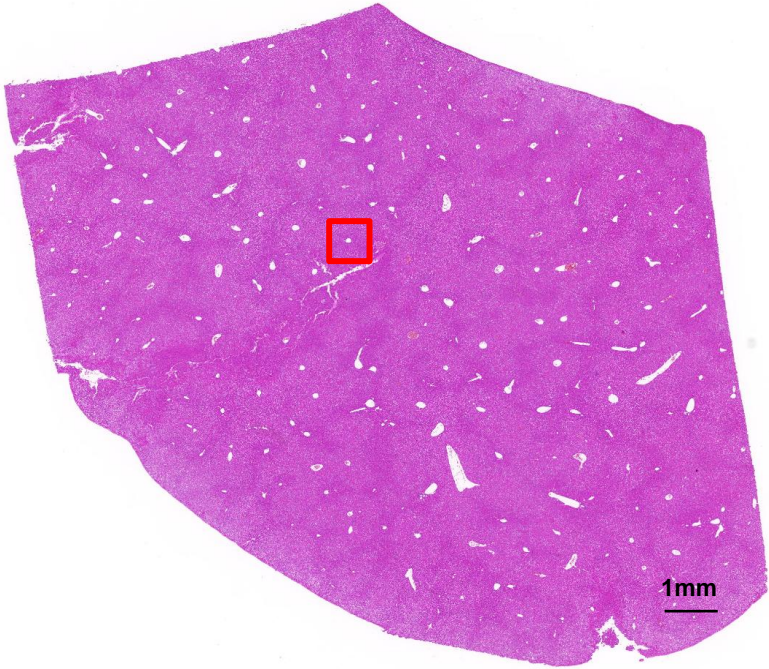

WT

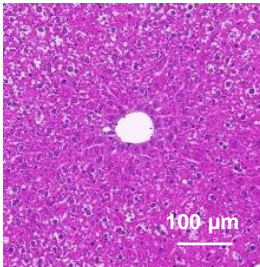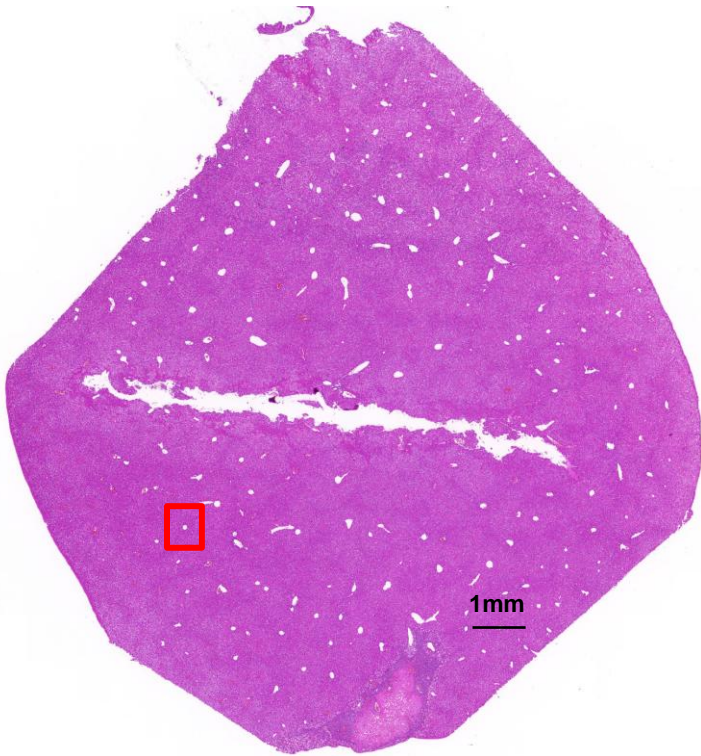

LKO

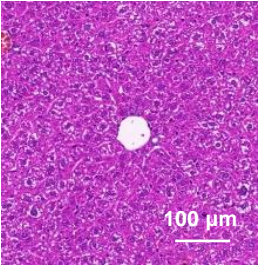

Supplement: Supplementary file 11 — Figure EV Source Data [file 44318_2025_525_MOESM11_ESM.zip › supp figures/Figure EV3/3D.pdf]

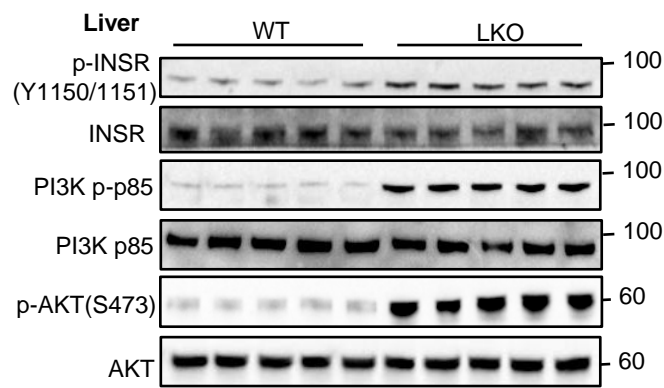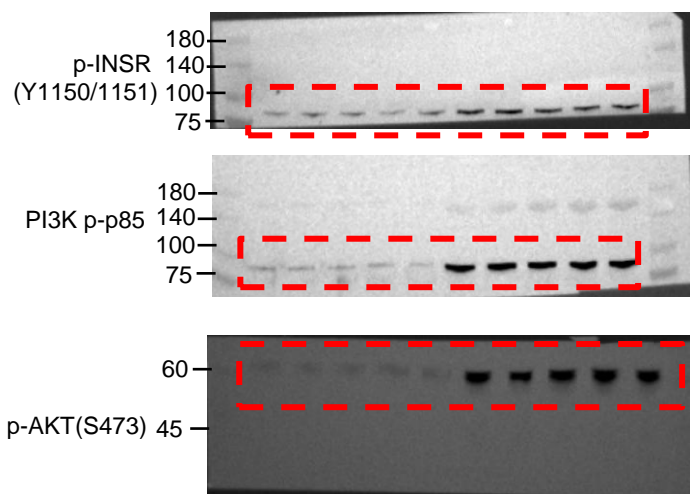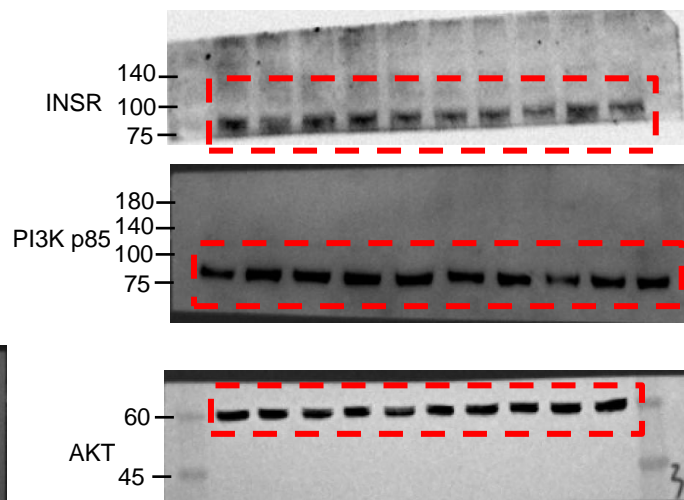

Supplement: Supplementary file 11 — Figure EV Source Data [file 44318_2025_525_MOESM11_ESM.zip › supp figures/Figure EV3/3I.pdf]

EV4D

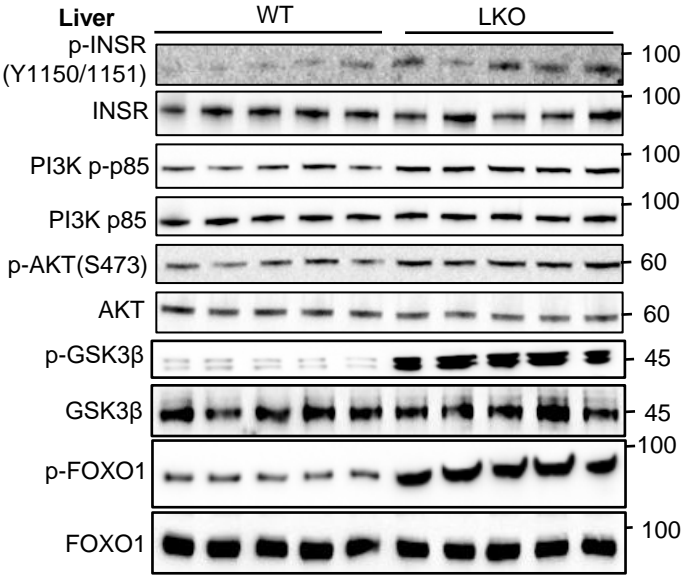

p-INSR(Y1150/1151)

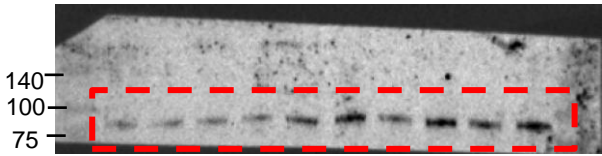

INSR

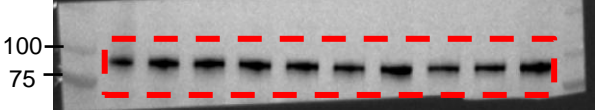

PI3K p-p85

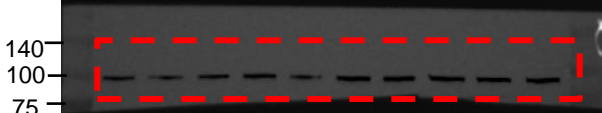

PI3K p85

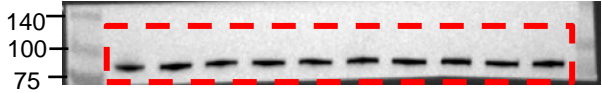

p-AKT(S473)

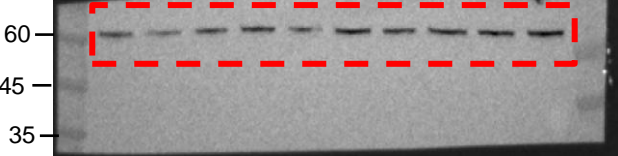

AKT

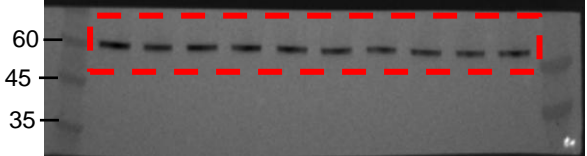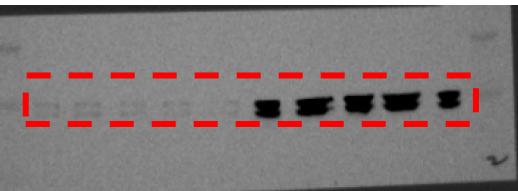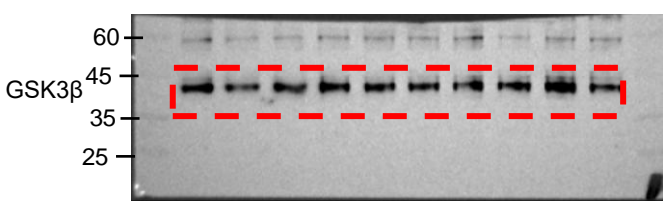

GSK3β

GSK3β

p-FOXO1

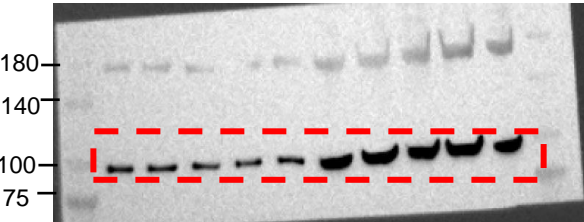

FOXO1

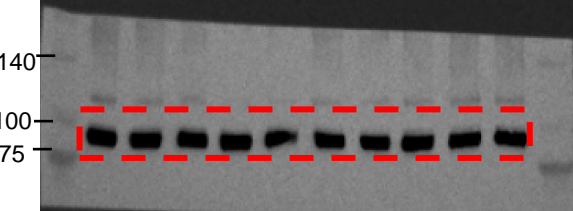

Supplement: Supplementary file 11 — Figure EV Source Data [file 44318_2025_525_MOESM11_ESM.zip › supp figures/Figure EV4/4D.pdf]

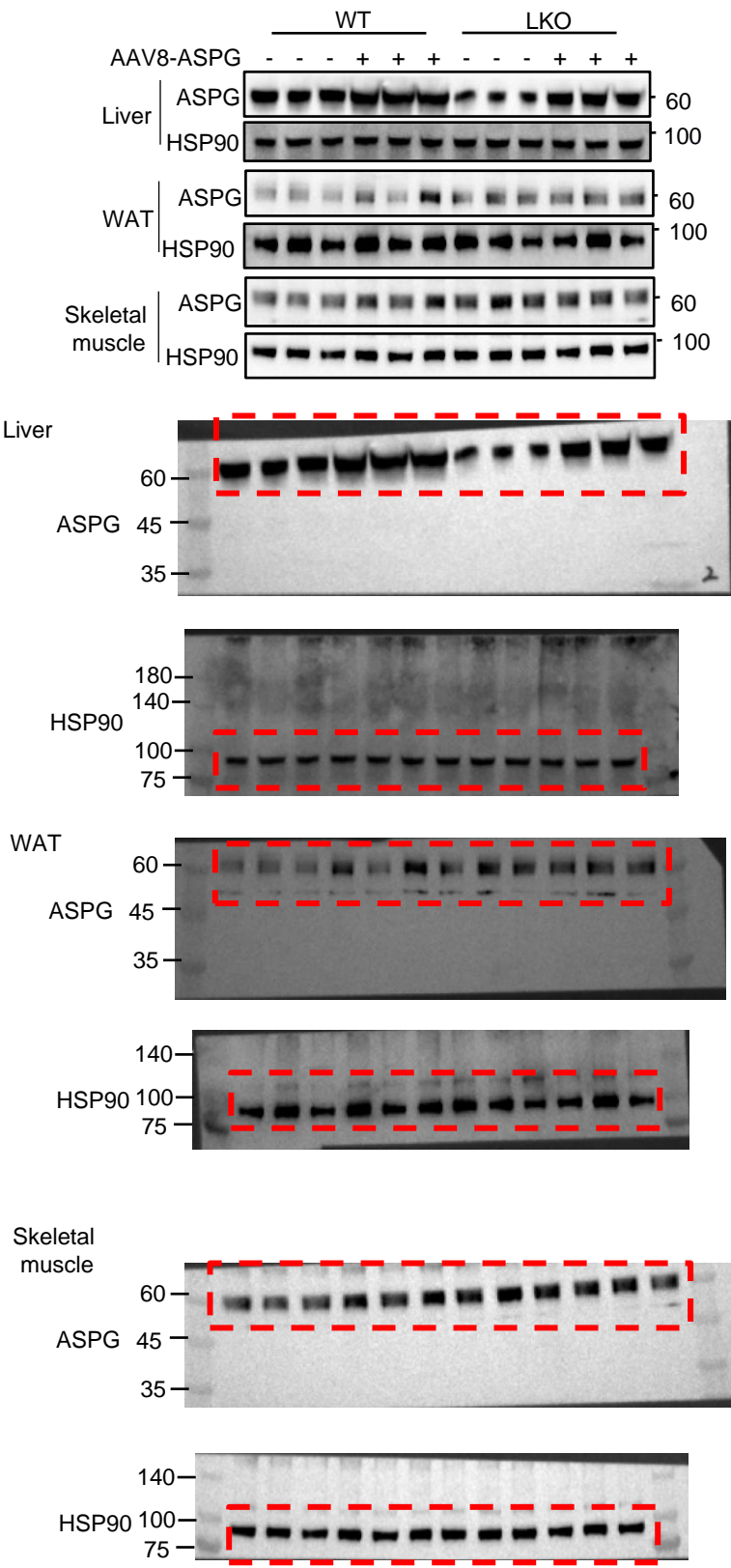

Supplement: Supplementary file 11 — Figure EV Source Data [file 44318_2025_525_MOESM11_ESM.zip › supp figures/Figure EV5/5C.pdf]

EV5H

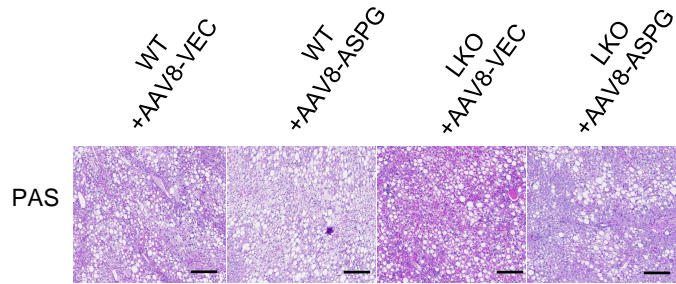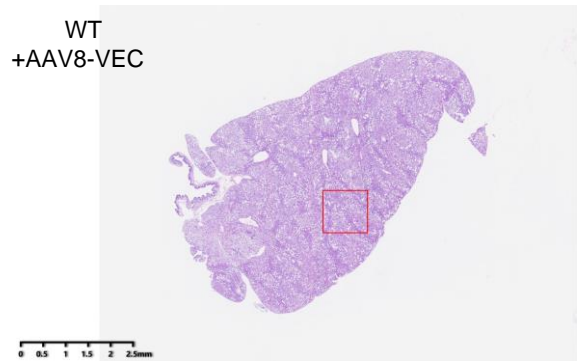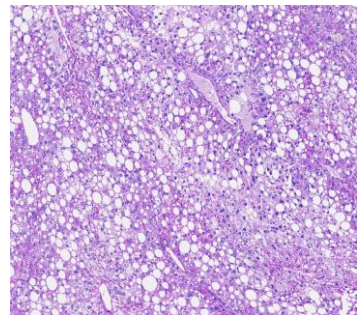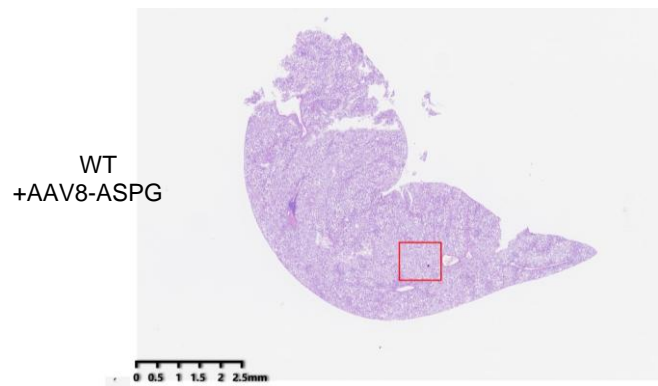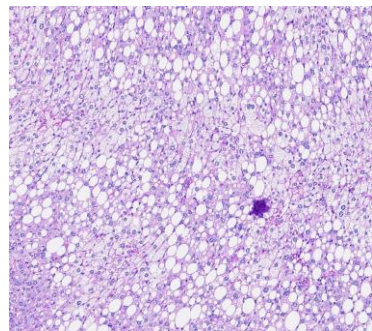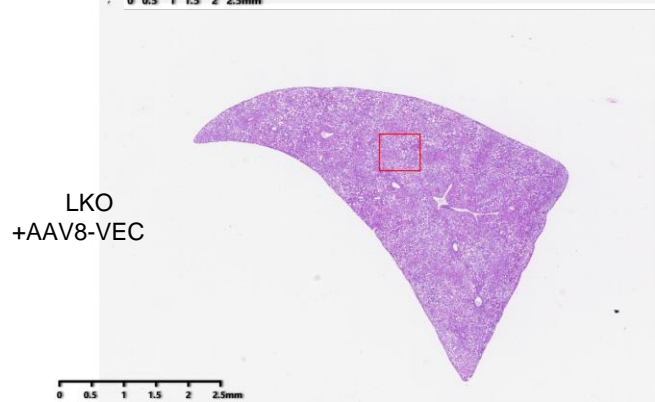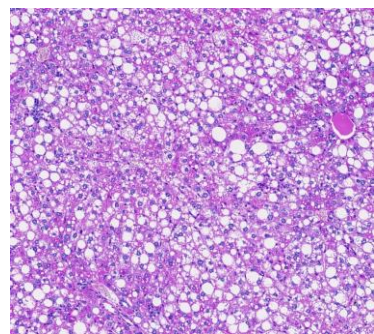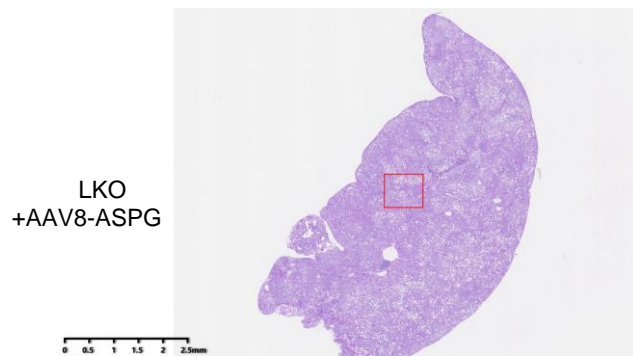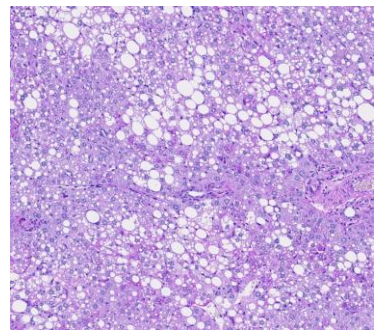

Supplement: Supplementary file 11 — Figure EV Source Data [file 44318_2025_525_MOESM11_ESM.zip › supp figures/Figure EV5/5H.pdf]

EV5M

WT  
+AAV8-VEC

WT  
+AAV8-ASPG

LKO  
+AAV8-VEC

LKO  
+AAV8-ASPG

F4/80

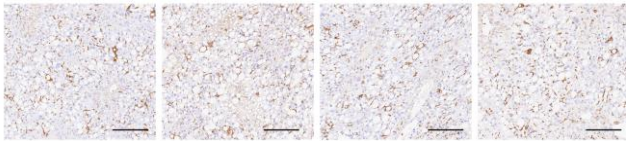

WT  
+AAV8-VEC

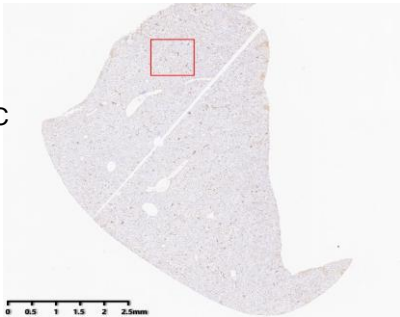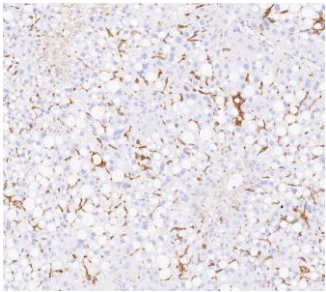

WT  
+AAV8-ASPG

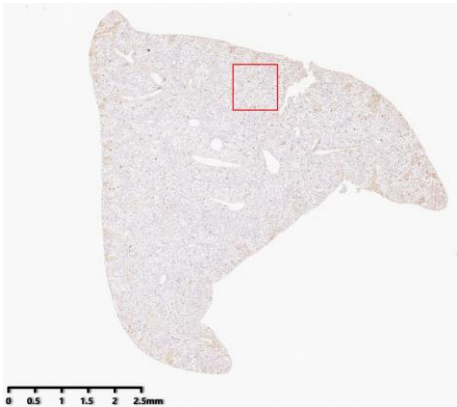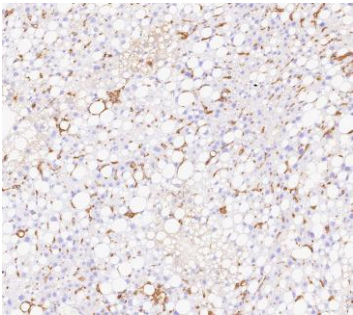

LKO  
+AAV8-VEC

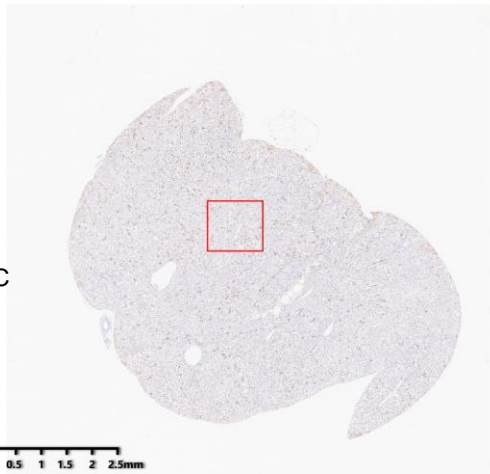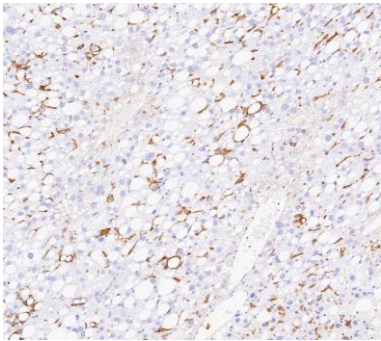

LKO  
+AAV8-ASPG

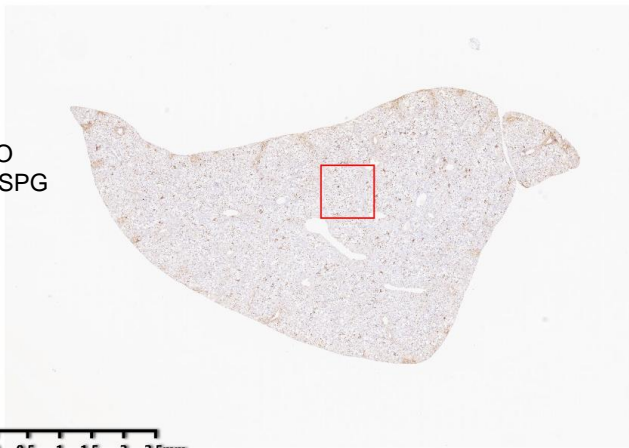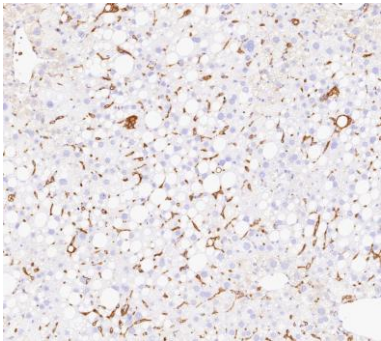

Supplement: Supplementary file 11 — Figure EV Source Data [file 44318_2025_525_MOESM11_ESM.zip › supp figures/Figure EV5/5M.pdf]

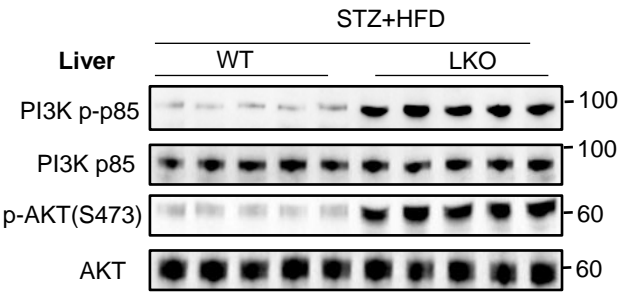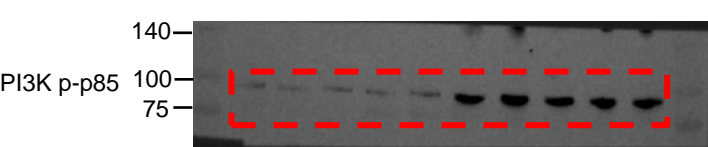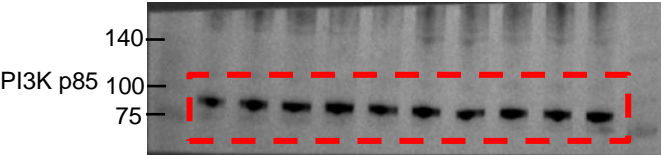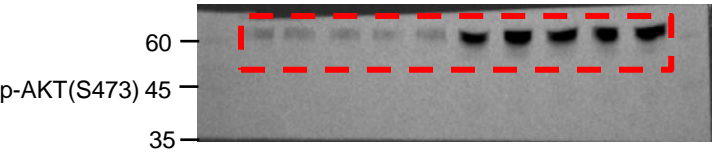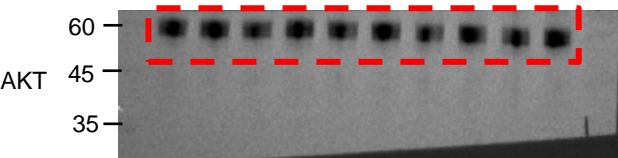

Supplement: Supplementary file 11 — Figure EV Source Data [file 44318_2025_525_MOESM11_ESM.zip › supp figures/Figure EV6/6F.pdf]

EV6G

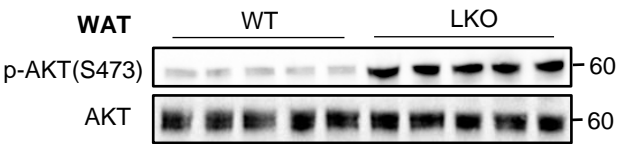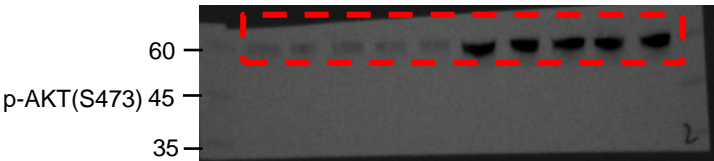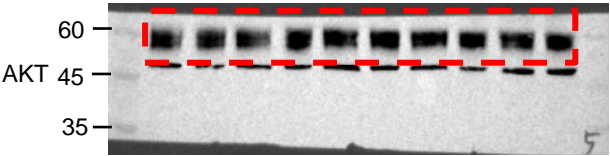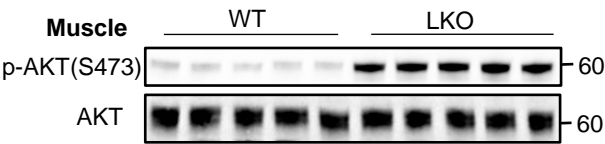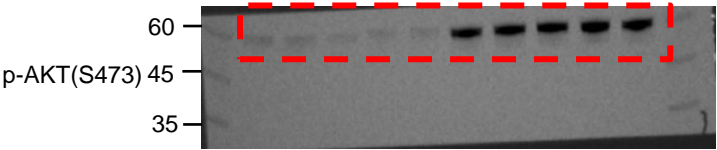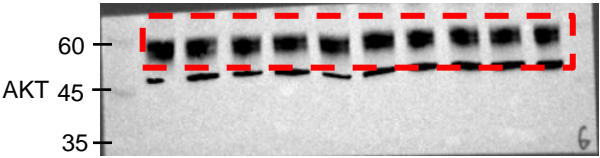

Supplement: Supplementary file 11 — Figure EV Source Data [file 44318_2025_525_MOESM11_ESM.zip › supp figures/Figure EV6/6G.pdf]

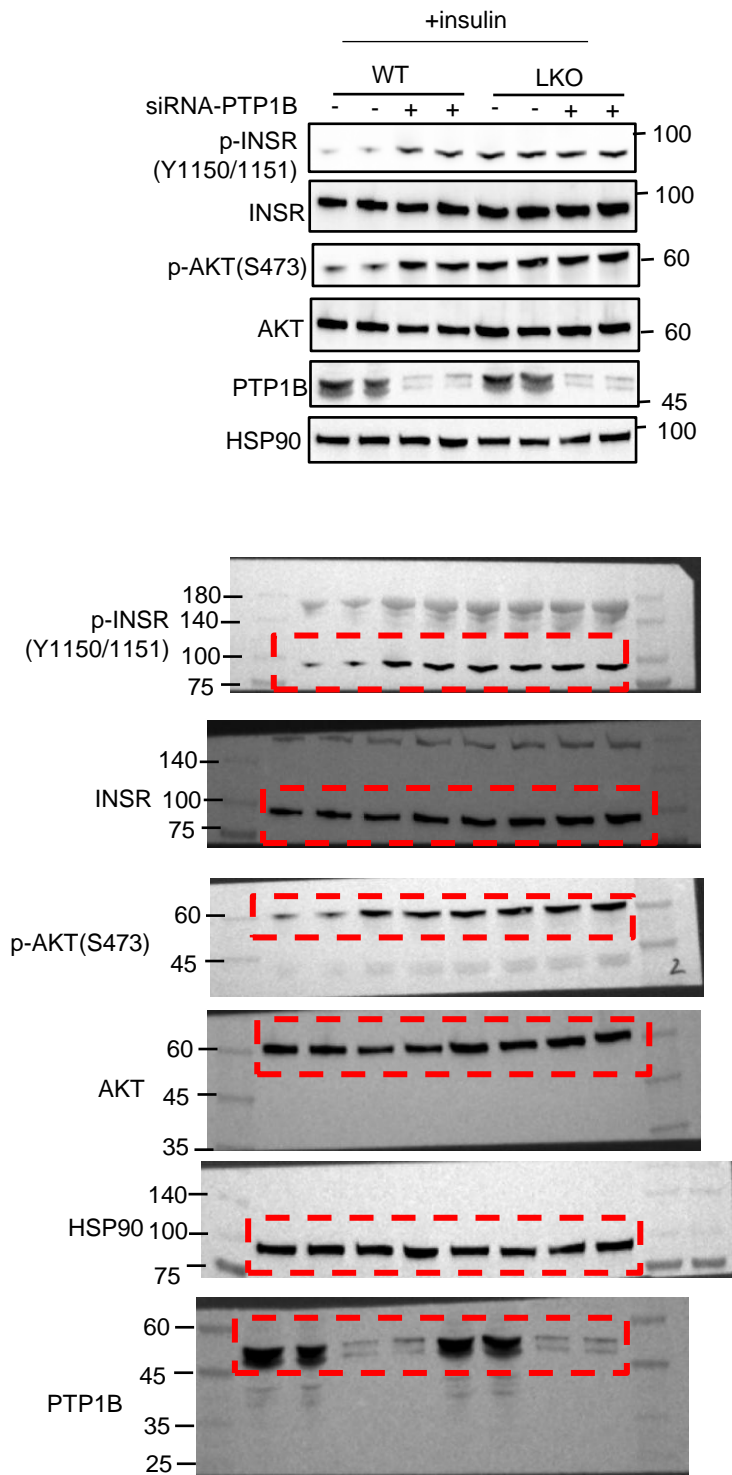

Supplement: Supplementary file 11 — Figure EV Source Data [file 44318_2025_525_MOESM11_ESM.zip › supp figures/Figure EV7/7H.pdf]

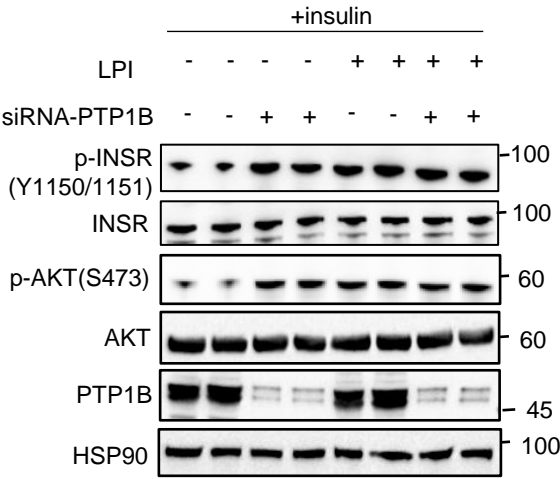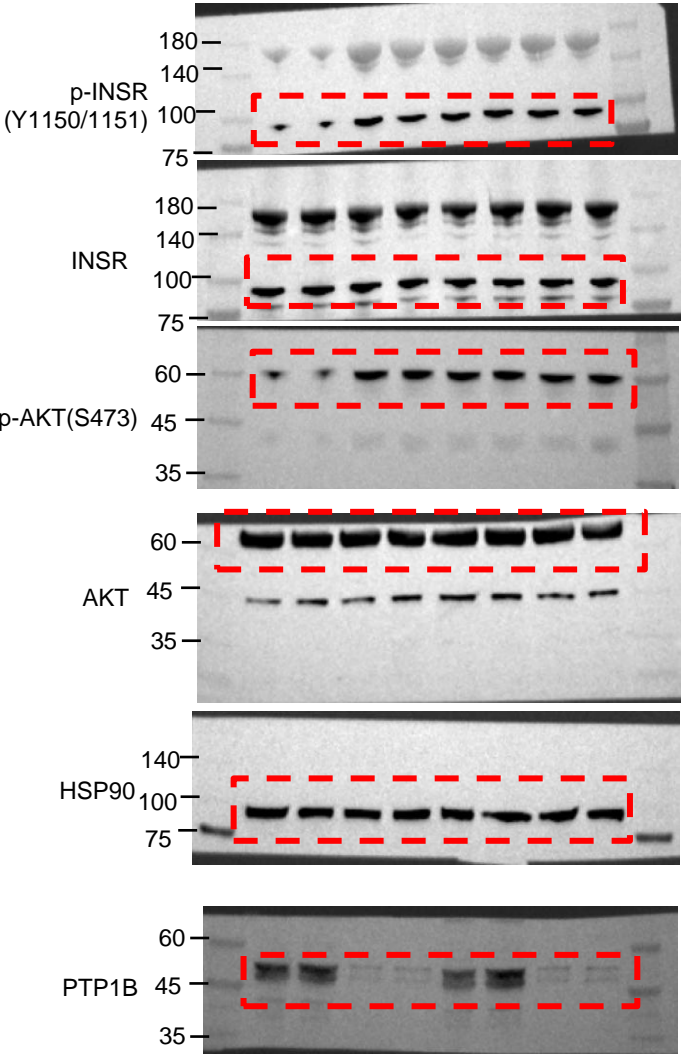

Supplement: Supplementary file 11 — Figure EV Source Data [file 44318_2025_525_MOESM11_ESM.zip › supp figures/Figure EV7/7I.pdf]

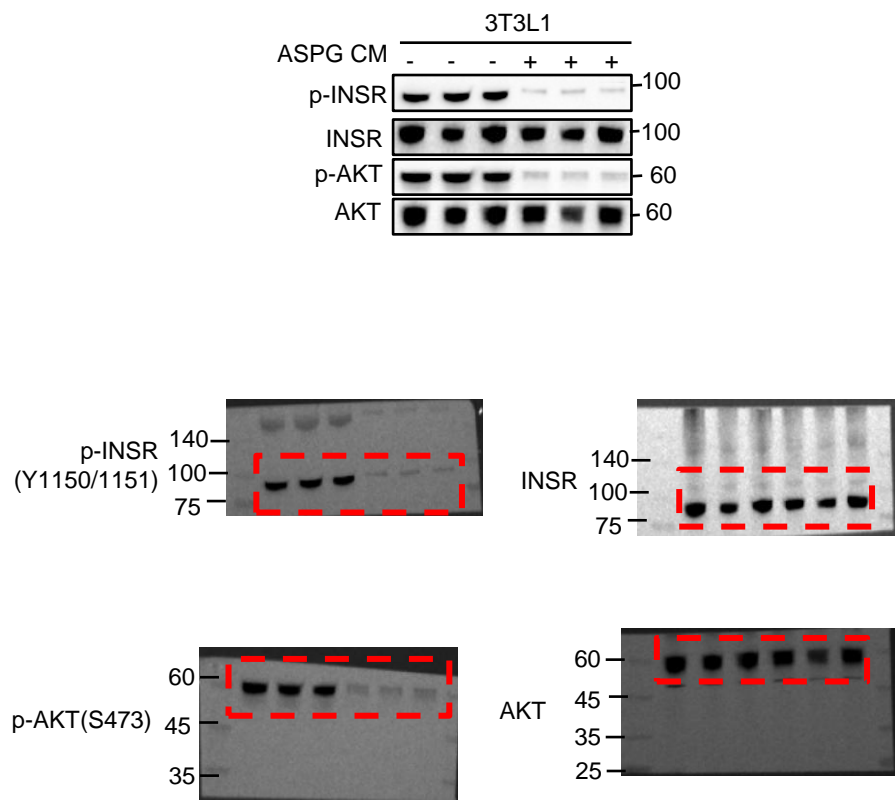

Supplement: Supplementary file 11 — Figure EV Source Data [file 44318_2025_525_MOESM11_ESM.zip › supp figures/Figure EV8/8D.pdf]
