# Supplementary material for: Hepatic ASPG-mediated lysophosphatidylinositol catabolism impairs insulin signal transduction
Source: EMBO J. 2025 Aug 4;44(18):5005–36. doi: 10.1038/s44318-025-00525-x (PMC12436650; doi:10.1038/s44318-025-00525-x)
Supplement: Supplementary file 12 — Expanded View Figures [file 44318_2025_525_MOESM12_ESM.pdf]

Expanded View Figures

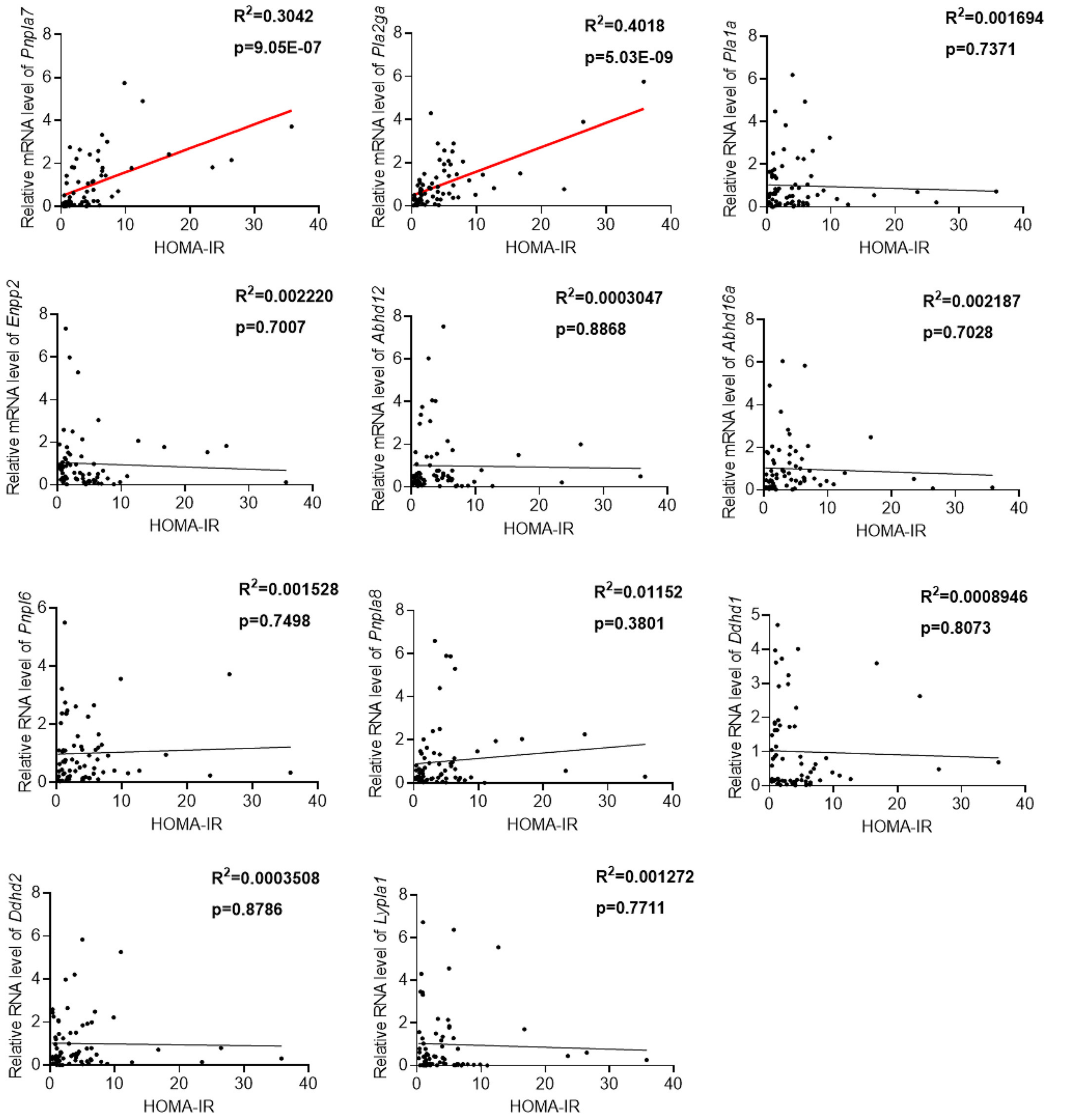

Figure EV1. The correlation between the mRNA levels of designated (lyso)phospholipases in the human liver samples and HOMA-IR.

Quantitative RT-PCR detected mRNA levels of designated genes in human liver specimen. $n = 69$, P values examined by the Pearson and Spearman correlation analysis. Source data are available online for this figure.

 © The Author(s)

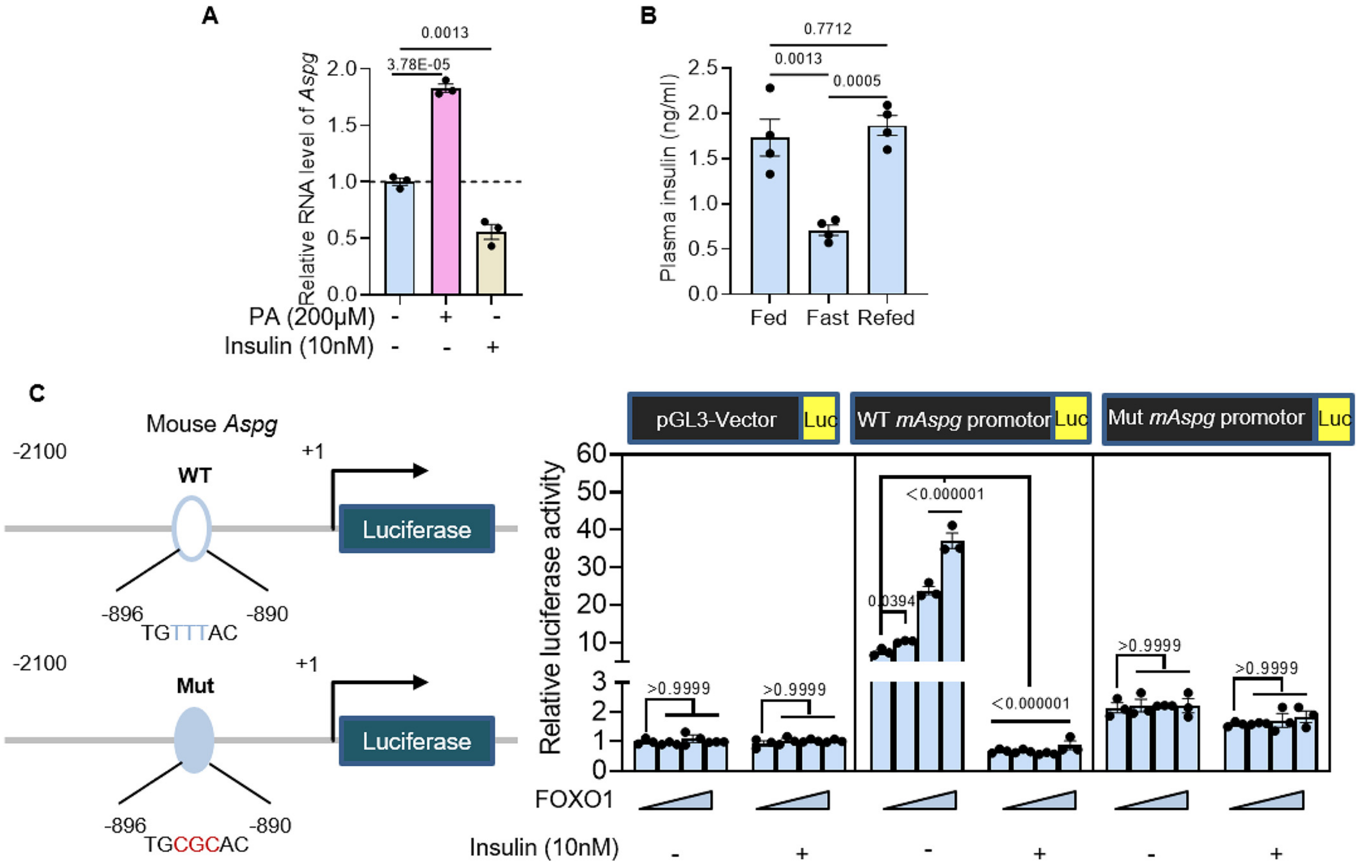

Figure EV2. Transcriptional factor FOXO1 upregulates hepatic *Aspg* expression.

(**A**) Primary hepatocytes isolated from the C57BL/6 mice were treated with 200 µM palmitate (PA) or 10 nM insulin for 24 h. *Aspg* mRNA level was measured by quantitative PCR ($n = 3$). (**B**) Plasma insulin level in feeding, 24 h-fasting and 2 h-refeeding C57BL/6 mice ($n = 4$). (**C**) Dual-luciferase reporter assay using mouse *Aspg* WT or mutant promoter to locate the sequence in the *Aspg* promoter responsible for the regulation of FOXO1. HEK293T cells were transfected indicated plasmids and treated with or without 10 nM insulin for 24 h before the assay ($n = 3$). Data are represented as mean ± SEM. Statistical analysis was performed by one-way ANOVA followed by Tukey's test for (**A–C**). Source data are available online for this figure.

© The Author(s)

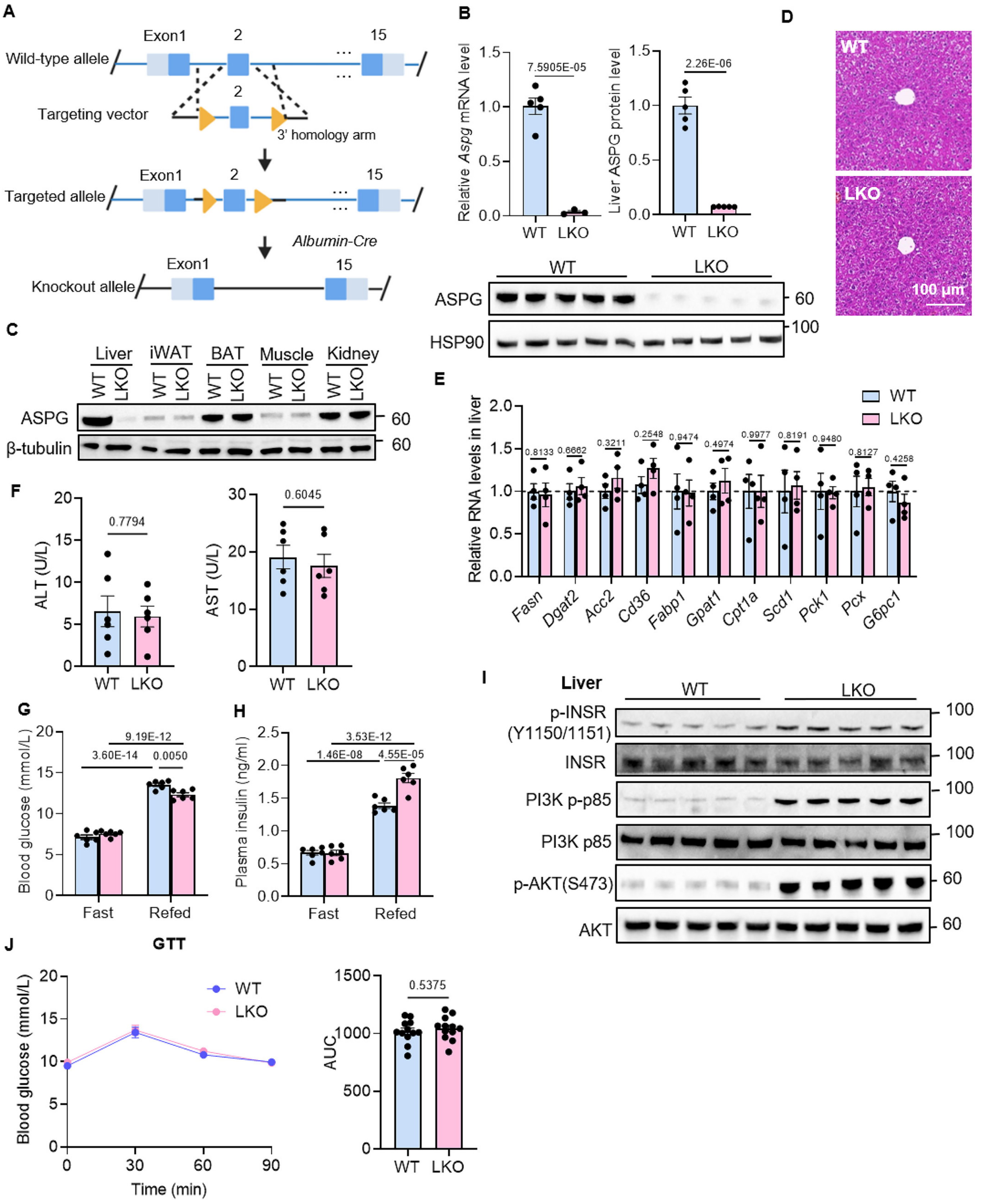
A
Wild-type allele
Exon1
2
15
Targeting vector
3' homology arm
Targeted allele
Albumin-Cre
Knockout allele
B
Relative Aspg mRNA level
7.5905E-05
Liver ASPG protein level
2.26E-06
WT LKO
ASPG
HSP90
C
Liver iWAT BAT Muscle Kidney
ASPG
β-tubulin
D
WT
LKO
100 μm
E
Relative RNA levels in liver
Fasn Dgat2 Acc2 Cd36 Fabp1 Gpat1 Cpt1a Scd1 Pck1 Pcx G6pc1
0.8133 0.6662 0.3211 0.2548 0.9474 0.4974 0.9977 0.8191 0.9480 0.8127 0.4258
F
ALT (U/L)
0.7794
AST (U/L)
0.6045
G
Blood glucose (mmol/L)
3.60E-14
9.19E-12
0.0050
Fast Refed
H
Plasma insulin (ng/ml)
1.46E-08
3.53E-12
4.55E-05
I
Liver
p-INSR (Y1150/1151)
INSR
PI3K p-p85
PI3K p85
p-AKT(S473)
AKT
J
GTT
Blood glucose (mmol/L)
Time (min)
AUC
0.5375

Figure EV3. The construction of hepatocyte *Aspg* knockout mice.

(**A**) Schematic diagram depicting the generation of hepatocyte-deficient *Aspg* mice. (**B**) Levels of the *Aspg* mRNA and protein in the liver samples from the chow diet feeding WT and *Aspg* LKO mice ($n = 5$ mice per group, male). (**C**) Representative western blotting showing the expression of ASPG in the liver, inguinal white adipose tissue (iWAT), brown adipose tissue (BAT), skeletal muscle and kidney. (**D**) The representative images of hematoxylin and eosin staining for liver tissues. Scale bars 100 µm. (**E**) The mRNA levels of lipogenesis, β-oxidation and gluconeogenesis genes in livers ($n = 4$ each genotype, male). (**F**) Serum alanine aminotransferase (ALT) (left) and aspartate aminotransferase (AST) (right) levels in chow diet feeding WT and *Aspg* LKO mice ($n = 5$ mice per group, male). (**G**, **H**) Levels of blood glucose (**G**) and plasma insulin (**H**) were measured in 24 h-fasting and 2 h-refeeding chow diet mice ($n = 6$ each genotype, male). (**I**) Western blot analysis for the liver tissues from the WT and *Aspg* LKO mice. The mice were fasted for 24 h followed by 2 h-refeeding to stimulate endogenous insulin secretion ($n = 5$ each genotype, male). (**J**) Glucose tolerance test (GTT) was measured for chow diet feeding mice. ($n = 12$ each genotype, male). Data are represented as mean ± SEM. Statistical analysis was performed by unpaired two-tailed Student's *t* test for (**B**, **E**, **F**, **J**), by two-way ANOVA followed by Tukey's test (**G**, **H**). Source data are available online for this figure.

© The Author(s)

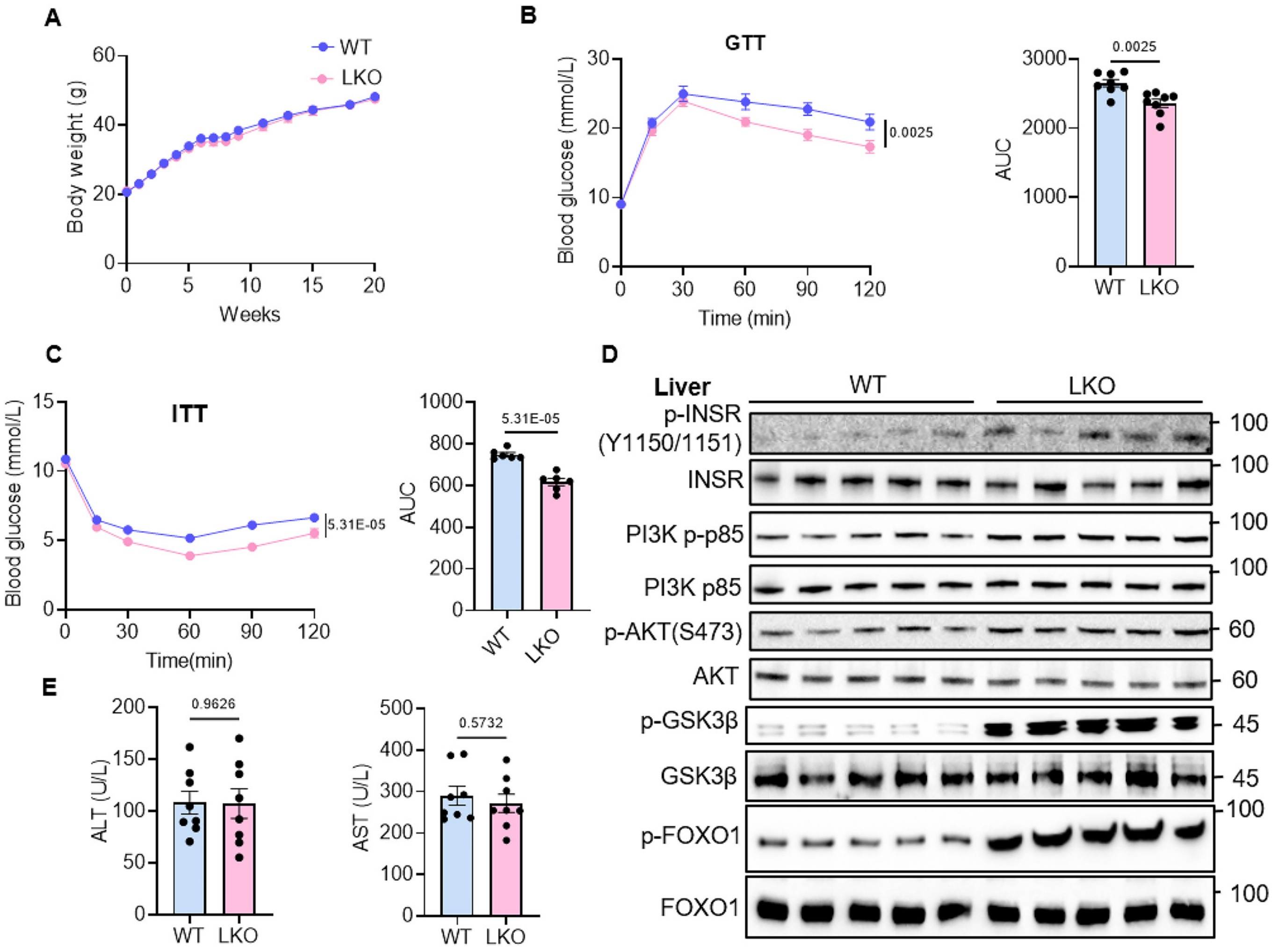

Figure EV4. Hepatic APSG deficiency improves glucose tolerance in obese female mice.

(**A**–**E**) Comparison of the phenotypic features of the WT and *Aspg* LKO female mice after a HFD for 20 weeks, including (**A**) body weight, (**B**) GTT, (**C**) ITT, (**D**) western blot analysis of essential markers of insulin signaling in the liver tissues, (**E**) serum ALT and AST levels ($n = 8$ each group for (**A**–**C**) and (**E**); $n = 5$ each group for (**D**), female). For all: Data are represented as mean ± SEM. Statistical analysis was performed by unpaired two-tailed Student's *t* test for (**B**, **C**, **E**). Source data are available online for this figure.

 © The Author(s)

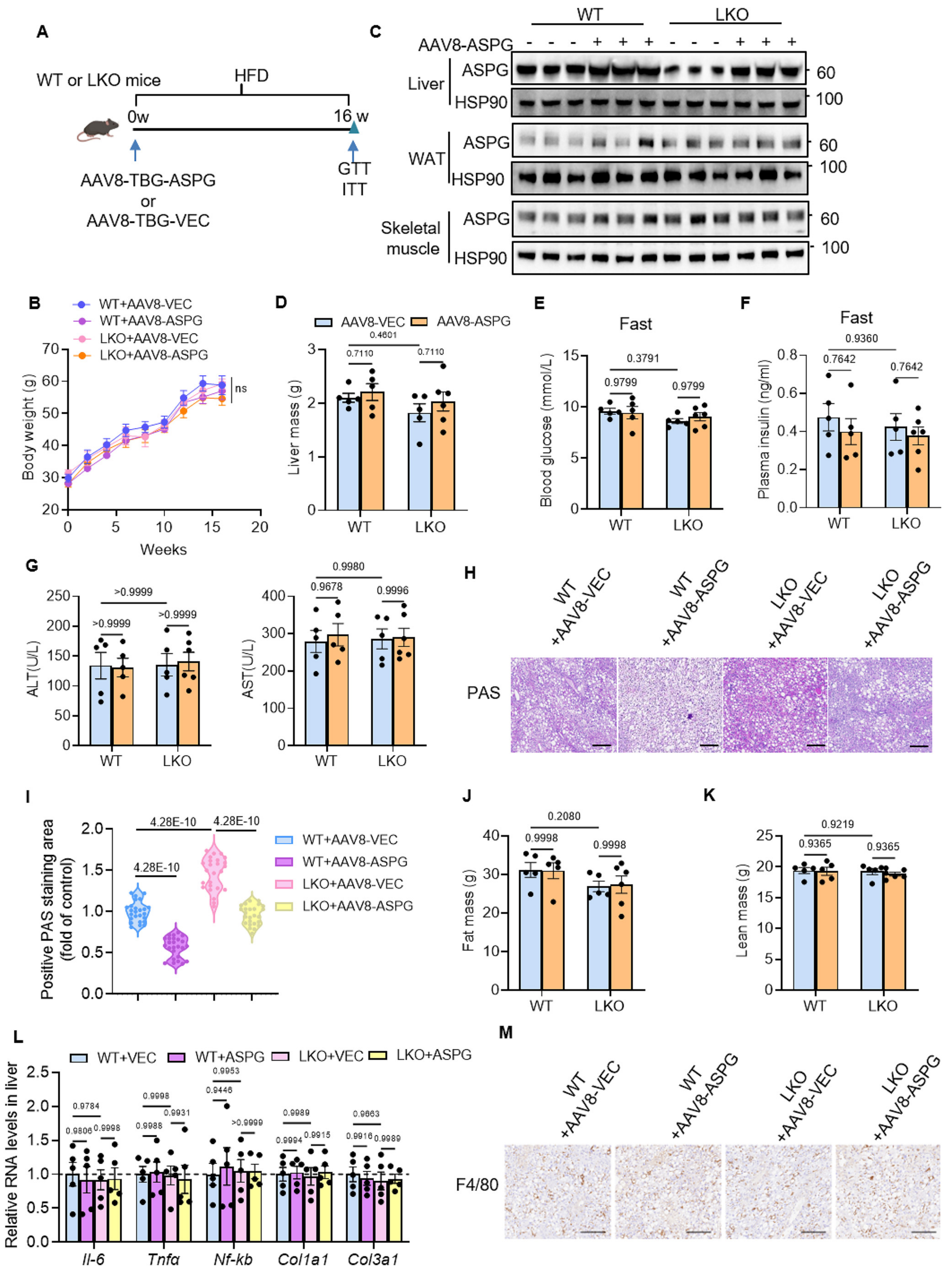
A
WT or LKO mice
HFD
0w
16 w
AAV8-TBG-ASPG
or
AAV8-TBG-VEC
GTT
ITT
C
WT
LKO
AAV8-ASPG
Liver
ASPG
HSP90
WAT
Skeletal muscle
60
100
B
WT+AAV8-VEC
WT+AAV8-ASPG
LKO+AAV8-VEC
LKO+AAV8-ASPG
Body weight (g)
Weeks
ns
D
AAV8-VEC
AAV8-ASPG
Liver mass (g)
0.4601
0.7110
0.7110
E
Fast
Blood glucose (mmol/L)
0.3791
0.9799
0.9799
F
Fast
Plasma insulin (ng/ml)
0.9360
0.7642
0.7642
G
ALT(U/L)
>0.9999
AST(U/L)
0.9980
0.9678
0.9996
H
PAS
WT +AAV8-VEC
WT +AAV8-ASPG
LKO +AAV8-VEC
LKO +AAV8-ASPG
I
Positive PAS staining area (fold of control)
4.28E-10
J
Fat mass (g)
0.2080
0.9998
K
Lean mass (g)
0.9219
0.9365
L
WT+VEC
WT+ASPG
LKO+VEC
LKO+ASPG
Relative RNA levels in liver
Il-6
Tnfα
Nf-kb
Col1a1
Col3a1
M
F4/80

Figure EV5. Hepatic overexpression of ASPG does not alter the progression of MASLD.

(**A**) Timeline of AAV-mediated hepatic *Aspg* expression in female WT and LKO mice. AAV-TBG-ASPG or AAV-TBG-Vector was injected to the WT or LKO mice. Then all the groups of mice were fed a HFD for 16 weeks. (**B**) The body weight of mice in (**A**) during HFD feeding (WT + AAV-VEC $n = 5$, WT + AAV-ASPG $n = 5$, LKO + AAV-VEC $n = 5$, LKO + AAV-ASPG $n = 6$, female). (**C**) Western blot analysis of ASPG expression in the livers, WATs and muscle for mice in (**A**) ($n = 3$). (**D–G**) Liver mass (**D**), fasting blood glucose (**E**) and plasma insulin (**F**) levels, serum ALT and AST levels (**G**) for mince in (**A**) (WT + AAV-VEC $n = 5$, WT + AAV-ASPG $n = 5$, LKO + AAV-VEC $n = 5$, LKO + AAV-ASPG $n = 6$, female). (**H**) The periodic acid-Schiff (PAS) staining showed the glycogen content in livers of mice in (**A**). Scale bar 200 µm. (**I**) Quantification of PAS staining. Total 25 microscope fields of view in each group have been quantified. (**J**, **K**) Fat mass (**J**) and lean mass (**K**) for mice in (**A**) (WT + AAV-VEC $n = 5$, WT + AAV-ASPG $n = 5$, LKO + AAV-VEC $n = 5$, LKO + AAV-ASPG $n = 6$, female). (**L**) Hepatic mRNA levels of inflammation- and fibrosis-related genes in livers of mice in (**A**) ($n = 5$ each group, female). (**M**) The representative F4/80-immunohistochemistry staining of liver sections for mice in (**A**). Scale bar 200 µm. For all: Data are represented as mean ± SEM. Statistical analysis was performed by two-way ANOVA followed by Tukey's test for (**B**, **D–G**, and **I–K**), by one-way ANOVA followed by Tukey's test for (**L**). Source data are available online for this figure.

 © The Author(s)

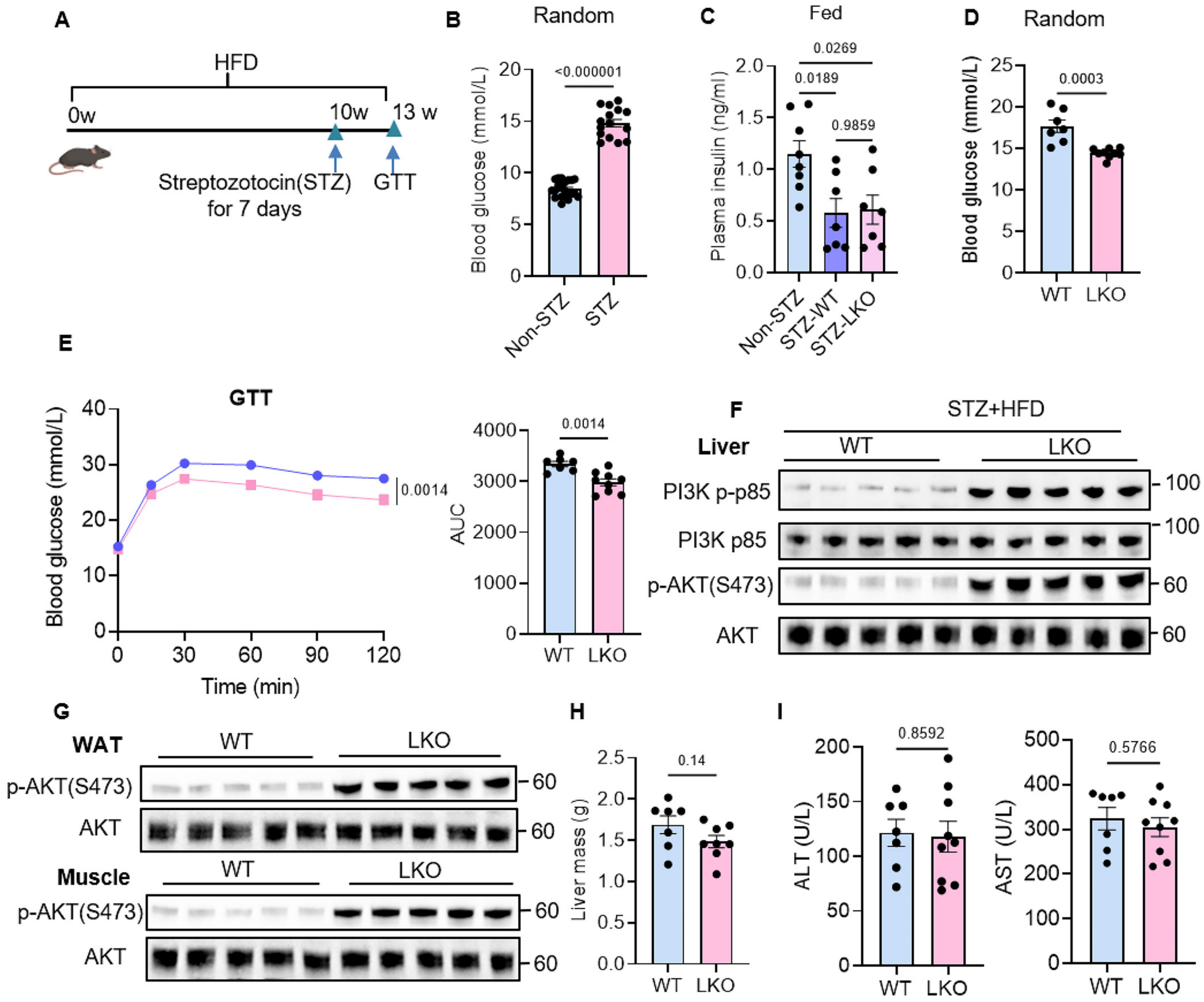

Figure EV6. Improved glucose tolerance in type 2 diabetic *Aspg* LKO mice.

(**A**) Timeline of streptozotocin (STZ)-induced type 2 diabetes mouse modeling. STZ (35 mg/kg body weight) were intraperitoneally injected to the HFD-fed male WT and *Aspg* LKO mice for 7 days. (**B**, **C**) Random blood glucose levels (**B**), Non-STZ $n = 21$, STZ $n = 15$, male) and fed plasma insulin levels (**C**), Non-STZ $n = 8$, STZ-WT $n = 7$, STZ-LKO $n = 7$, male) were measured in non-STZ, STZ or STZ-WT and STZ-*Aspg* LKO mice. (**D**) Levels of random blood glucose measured two weeks after STZ injection (STZ-WT $n = 7$, STZ-LKO $n = 9$, male). (**E**) GTT was performed two weeks after STZ injection (STZ-WT $n = 7$, STZ-LKO $n = 9$, male). The area under the curve (AUC) was used to quantify the GTT results. (**F**, **G**) Western blot analysis of essential markers of insulin signaling in the STZ/HFD mice liver, WAT and muscle ($n = 5$ each genotype, male). (**H**) Liver mass was measured in STZ/HFD mice (STZ-WT $n = 7$, STZ-LKO $n = 8$, male). (**I**) Serum ALT and AST levels in STZ/HFD mice ((STZ-WT $n = 7$, STZ-LKO $n = 9$, male). For all: Data are represented as mean ± SEM. Statistical analysis was performed by unpaired two-tailed Student's t test for (**B**, **D**, **E**, **H**, **I**), by one-way ANOVA followed by Tukey's test for (**C**). Source data are available online for this figure.

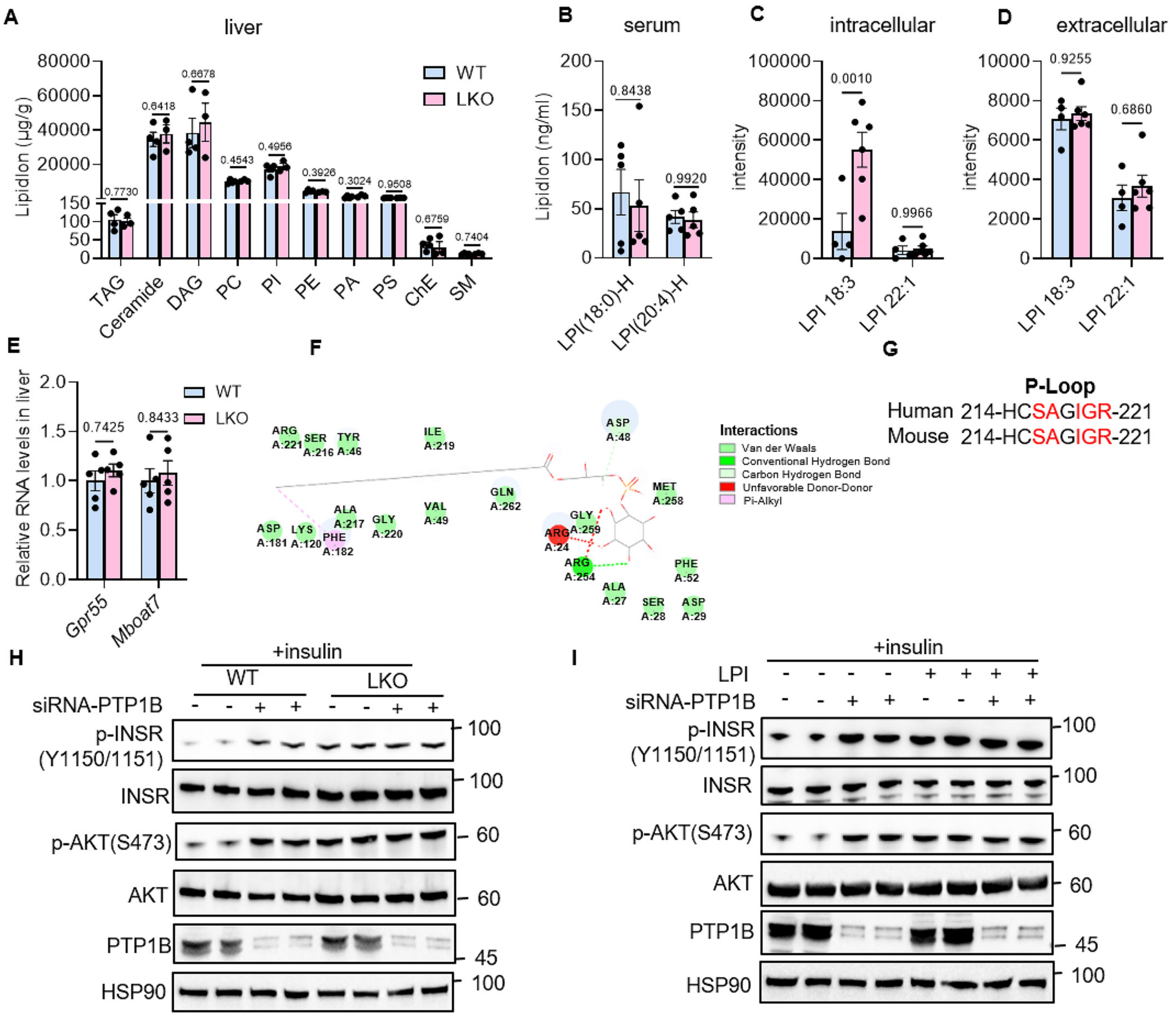

Figure EV7. Increased LPIs upon *Aspg* deficiency in hepatocytes inhibit PTP1B activity.

(**A**) Quantity of the 10 lipid species in liver tissues from the WT and *Aspg* LKO mice that were fed a HFD for 20 weeks (WT $n = 4$, LKO $n = 3$, female). (**B**) Quantity of LPIs in serum from the mice treated as in (**A**) ($n = 5$ each genotype, female). (**C**, **D**) The relative quantity of the intracellular (**C**) and extracellular LPI levels (**D**) in primary hepatocytes. The hepatocytes were isolated from the chow diet-fed male WT and *Aspg* LKO mice (WT $n = 4$, LKO $n = 6$). (**E**) Relative mRNA levels of *Gpr55* and *Mboat7* in livers from the WT and LKO mice. The mice were fed a HFD for 16 weeks ($n = 5$ each group, male). (**F**) Molecular docking revealed interaction of LPI 18:0 with human PTP1B protein. (**G**) The sequence alignment of human and mouse PTP1B proteins. The amino acid residues interacting with LPI were indicated in red. (**H**) The siRNA against PTP1B was used to knockdown PTP1B in the primary hepatocytes. Western blot analysis of the indicated proteins was performed after insulin (10 nM) treatment for 15 min. (**I**) The siRNA against PTP1B was used to knockdown PTP1B in the primary hepatocytes followed by LPI (10 μM) treating the cells for 12 h. Western blot analysis was performed after insulin (10 nM) treatment for 15 min. For all: Data are represented as mean ± SEM. Statistical analysis was performed by unpaired two-tailed Student's *t* test for (**A–E**). Source data are available online for this figure.

 © The Author(s)

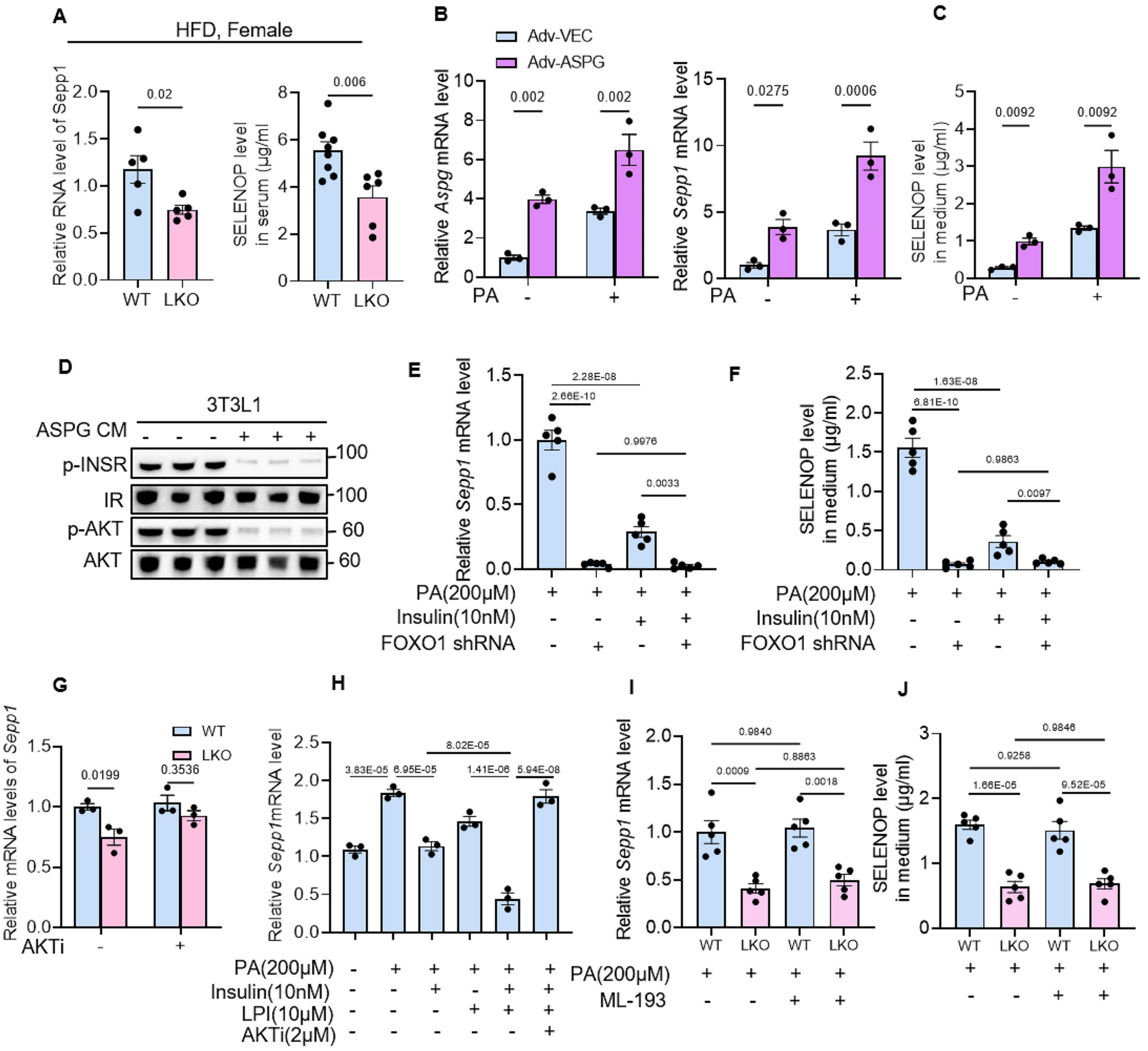

Figure EV8. ASPG regulates *Sepp1* expression.

(**A**) Relative mRNA levels of *Sepp1* in livers (left, $n = 5$ female) and SELENOP protein levels in serum (right, WT $n = 8$, LKO $n = 6$, female) from the WT and *Aspg* LKO mice that were fed a HFD for 20 weeks. (**B**) Adenoviruses expressing *Aspg* (Adv-ASPG) or the vector control (Adv-GFP) infected the primary hepatocytes followed by with or without palmitate (200 μM) treating the cells for 24 h. Then the mRNA levels of *Aspg* and *Sepp1* were determined by quantitative real-time PCR ($n = 3$ each group). (**C**) The SELENOP protein levels in the culture medium of cells in (**B**) ($n = 3$ each group). (**D**) Conditional medium collected from primary hepatocytes overexpressing Adv-ASPG after palmitic acid treatment for 24 h was applied to 3T3L1 adipocytes for 48 h. Western blot analysis of the phosphorylation and total protein levels of INSR and AKT in 3T3L1 adipocytes ($n = 3$). (**E, F**) Lentiviral *Foxo1* shRNA infected mouse primary hepatocytes followed by treatment with palmitate (36 h), insulin (24 h). *Sepp1* mRNA (**E**) and SELENOP protein levels (**F**) were measured ($n = 5$ each group). (**G**) Relative mRNA levels of *Sepp1* in the primary hepatocytes isolated from the WT and *Aspg* LKO mice. The cells were treated with or without AKTi (2 μM) for 2 h before the assay ($n = 3$ each group). (**H**) Relative mRNA levels of *Sepp1* in the mouse primary hepatocytes. The cells were treated with palmitate (36 h), insulin (24 h), LPI (12 h) and AKTi (2 h) as indicated following by a quantitative RT-PCR analysis ($n = 3$). (**I, J**) Mouse primary hepatocytes were treated by PA (36 h) and ML-193 (6 h) prior to the *Sepp1* mRNA (**I**) and SELENOP protein assay (**J**). $n = 5$ each group. For all: Data are represented as mean ± SEM. Statistical analysis was performed by unpaired two-tailed Student's *t* test for (**A**), by two-way ANOVA followed by Tukey's test for (**B, C, G**), by one-way ANOVA followed by Tukey's test for (**E, F, H–J**). Source data are available online for this figure.

© The Author(s)

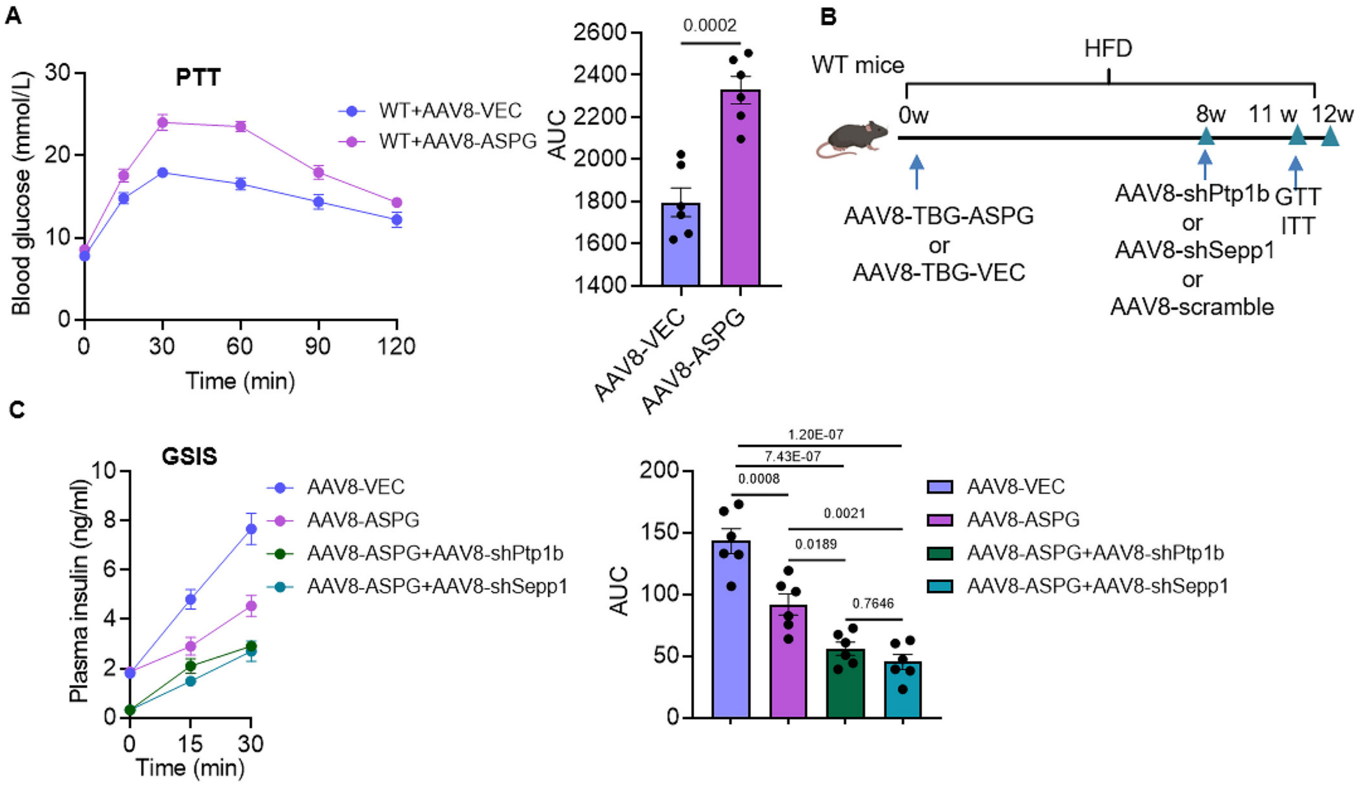

Figure EV9. Knocking down *Ptp1b* or *Sepp1* improves glucose metabolic homeostasis in Aspg-overexpression mice.

(**A**) *Aspg*-overexpressing mice showed increased gluconeogenesis in pyruvate tolerance test (PTT) after 8-week HFD feeding. $n = 6$ each group, male. (**B**) Timeline of AAV-mediated hepatic *Aspg* expression followed by *Ptp1b* or *Sepp1* knockdown. (**C**) The serum insulin levels were measured during the first 30 min of GTT assay. $n = 6$ each group, male. For all: Data are represented as mean ± SEM. Statistical analysis was performed by unpaired two-tailed Student's *t* test for (**A**), by one-way ANOVA followed by Tukey's test for (**C**). Source data are available online for this figure.
